# Efficacy of alternative or adjunctive measures to conventional non-surgical and surgical treatment of peri-implant mucositis and peri-implantitis: a systematic review and meta-analysis

**DOI:** 10.1186/s40729-021-00388-x

**Published:** 2021-11-15

**Authors:** Ausra Ramanauskaite, Tobias Fretwurst, Frank Schwarz

**Affiliations:** 1 Department of Oral Surgery and Implantology, Johann Wolfgang Goethe-University Frankfurt, Carolinum, 60596 Frankfurt am Main, Germany; 2 Department of Oral- and Maxillofacial Surgery, Medical Center, Faculty of Medicine, University of Freiburg, University of Freiburg, Frankfurt, Germany; 3 Department of Oral Surgery and Implantology, Goethe University, Carolinum, Frankfurt, Germany

**Keywords:** Systematic review, Peri-implant disease, Treatment, Dental implant

## Abstract

**Purpose:**

To evaluate the efficacy of alternative or adjunctive measures to conventional non-surgical or surgical treatment of peri-implant mucositis and peri-implantitis.

**Material and methods:**

Prospective randomized and nonrandomized controlled studies comparing alternative or adjunctive measures, and reporting on changes in bleeding scores (i.e., bleed0ing index (BI) or bleeding on probing (BOP)), probing depth (PD) values or suppuration (SUPP) were searched.

**Results:**

Peri-implant mucositis: adjunctive use of local antiseptics lead to greater PD reduction (weighted mean difference (WMD) = − 0.23 mm; *p* = 0.03, respectively), whereas changes in BOP were comparable (WMD = − 5.30%; *p* = 0.29). Non-surgical treatment of peri-implantitis: alternative measures for biofilm removal and systemic antibiotics yielded higher BOP reduction (WMD = − 28.09%; *p* = 0.01 and WMD = − 17.35%; *p* = 0.01, respectively). Surgical non-reconstructive peri-implantitis treatment: WMD in PD amounted to − 1.11 mm favoring adjunctive implantoplasty (*p* = 0.02). Adjunctive reconstructive measures lead to significantly higher radiographic bone defect fill/reduction (WMD = 56.46%; *p* = 0.01 and WMD = − 1.47 mm; *p* = 0.01), PD (− 0.51 mm; *p* = 0.01) and lower soft-tissue recession (WMD = − 0.63 mm; *p* = 0.01), while changes in BOP were not significant (WMD = − 11.11%; *p* = 0.11).

**Conclusions:**

Alternative and adjunctive measures provided no beneficial effect in resolving peri-implant mucositis, while alternative measures were superior in reducing BOP values following non-surgical treatment of peri-implantitis. Adjunctive reconstructive measures were beneficial regarding radiographic bone-defect fill/reduction, PD reduction and lower soft-tissue recession, although they did not improve the resolution of mucosal inflammation.

**Supplementary Information:**

The online version contains supplementary material available at 10.1186/s40729-021-00388-x.

## Introduction

Peri-implant diseases were defined during the 2017 World Workshop as biofilm‐associated pathological conditions affecting osseointegrated dental implants, and they were further classified into peri-implant mucositis and peri-implantitis [1–3]. Peri-implant mucositis is characterized by inflammation in the soft tissue compartment, whereas peri-implantitis also features loss of the implant-supporting bone [1–3]. It is assumed that untreated peri-implant mucositis is the precursor to peri-implantitis [4]. The onset of peri-implantitis was shown to occur early on, and its progression was characterized by a nonlinear, accelerating pattern that, in the absence of therapy, may ultimately lead to implant loss [5]. Numerous cross-sectional studies have recently reported on the high prevalence of peri-implant diseases, pointing to their common appraisal in daily clinical practice [6–9].

There is evidence from experimental clinical studies that peri-implant mucositis is a reversible condition if adequate bacterial plaque control is implemented [10, 11]. Non-surgical therapy in conjunction with oral hygiene reinforcement is considered a standard care treatment for managing peri-implant mucositis [1, 12]. At peri-implantitis sites, in contrast, non-surgical mechanical treatment alone or with adjunctive (i.e., local antibiotics, antimicrobial photodynamic therapy—aPDT) or alternative measures (e.g., air abrasive devices, erbium-doped yttrium aluminum garnet—Er:YAG laser monotherapy), has demonstrated only limited efficacy in obtaining disease resolution, indicating the necessity of surgical therapy in a majority of the cases [12, 13].

Recently, numerous surgical treatment protocols have been advocated for treatment of peri-implantitis using various surface decontamination approaches, along with resective measures (e.g., apical flap, osteoplasty, implantoplasty), reconstructive measures (e.g., bone fillers/autografts, guided bone regeneration), or a combination thereof (referred to as combined therapy) [13, 14]. Nonetheless, the reported efficacy of different surgical treatment approaches in arresting further disease progression varied considerably [15–20].

Currently, it remains unclear which interventions are most effective for the management of peri-implant diseases. Therefore, the aim of this systematic review and meta-analysis was to address the following focused question: In patients with peri-implant mucositis or peri-implantitis, what is the efficacy of non-surgical and surgical treatment with alternative or adjunctive measures on changing signs of inflammation compared to conventional non-surgical and surgical treatments alone?

## Materials and methods

The review protocol was developed and structured according to the PRISMA (Preferred Re-porting Items for Systematic Review and Meta-Analyses) Statement [21]. The review was registered in PROSPERO, an international prospective register of systematic reviews (CRD42021247402).

### Focused question

The focused question serving for literature search was structured according to the PICO format: “In patients with peri-implant mucositis and peri-implantitis, what is the efficacy of non-surgical (i.e., referring to peri-implant mucositis and peri-implantitis) and surgical (i.e., referring to peri-implantitis) treatments with alternative or adjunctive measures on changing signs of inflammation compared with conventional non-surgical and surgical treatments alone?”.

#### Population

Patients with peri-implant mucositis and peri-implantitis based on case definitions used in respective studies.

#### Intervention

Alternative (for biofilm removal) or adjunctive (local or systemic application of adjunctive antiseptic/antibiotic or reconstructive/resective therapy) measures to non-surgical and surgical treatments of peri-implant mucositis or peri-implantitis.

#### Comparison

Conventional measures for non-surgical and surgical treatments.

#### Outcome: primary outcomes

Changes in bleeding scores (i.e., bleeding index (BI), modified BI (mBI), sulcus bleeding index (SBI), or bleeding on probing (BOP), suppuration (SUPP), and probing depth (PD) values; *secondary outcomes:* changes in peri-implant mucosal level (ML) and radiographic marginal bone levels (RBL), radiographic defect fill (RDF).

*Study design:* Prospective randomized controlled (RCT), or nonrandomized controlled (CCT) studies (split-mouth or parallel group designs).

### Study inclusion and exclusion criteria

#### Inclusion criteria:

1. Studies on peri-implant mucositis: Studies comparing alternative (i.e., for biofilm removal) or adjunctive measures (i.e., adjunctive antiseptic/antibiotic oral or systemic application) to conventional non-surgical (i.e., mechanical/ultrasonic debridement) treatment with at least 3 months of follow-up.
2. Studies on non-surgical treatment of peri-implantitis: Studies comparing alternative (i.e., for biofilm removal) or adjunctive measures (i.e., adjunctive antiseptic/antibiotic oral or systemic application) to conventional non-surgical (i.e., mechanical/ultrasonic debridement with or without chlorhexidine (CHX) irrigation) treatment with at least 6 months of follow-up.
3. Studies on surgical treatment of peri-implantitis: Studies comparing adjunctive measures (i.e., adjunctive measures for implant surface decontamination, resective therapy by means of implantoplasty or reconstructive approaches) to conventional surgical treatment (i.e., access flap surgery) with at least 6 months of follow-up.
4. Studies reporting on clinical changes in bleeding scores (i.e., BI/BOP), SUPP and/or PDs, following non-surgical (referring to peri-implant mucositis and peri-implantitis) or surgical (referring to peri-implantitis) treatments in respective groups.
5. Studies providing case definitions of peri-implant mucositis and peri-implantitis.
6. Studies with a minimum of 10 patients (5 per treatment group).

The literature search was restricted to English language.

Exclusion criteria:

1. Inclusion of less than five patients per treatment group.
2. Lack of case definition.
3. Lack of clinical data on the changes in BOP/BI, PD or SUPP.

### Information source and search

Two electronic databases (MEDLINE (via PubMed) and The Cochrane Library) were searched for relevant articles published until 1^st^ April 2021. The search filter ‘humans’ was applied. Electronic search was complemented by a hand search of the following journals:

Clinical Implant Dentistry and Related Research; Clinical Oral Implants Research; International Journal of Oral and Maxillofacial Implants; Journal of Clinical Periodontology; Journal of Periodontology.

The combination of the following key words (i.e., Medical Subject Headings MeSH) and free text terms included:

“treatment” OR “nonsurgical treatment” OR “non-surgical treatment” OR “surgical treatment” OR “regenerative treatment” OR “augmentative treatment” OR “respective treatment” OR “reconstructive treatment” OR “therapy” OR “nonsurgical therapy” OR “non surgical therapy” OR “surgical therapy” OR “regenerative therapy” OR “augmentative therapy” OR “resective therapy” OR “reconstructive therapy” OR “antiseptic treatment” OR “antibiotic treatment” OR “adjunctive treatment” OR “antiseptic therapy” OR “antibiotic therapy” OR “adjunctive therapy”

AND

“peri-implant disease” OR “periimplant disease” OR “peri-implant infection” OR “periimplant infection” OR “mucositis” (MeSH) OR “peri-implant mucositis” OR “periimplant mucositis” OR “Periimplantitis” (MeSH) OR “peri-implantitis”.

### Study selection

During the first literature-selection stage, according to the defined inclusion criteria, the titles and abstracts of all identified studies were screened for eligibility by two independent reviewers (A.R. and F.S.). In the second stage, the full texts of potentially eligible articles were reviewed and evaluated according to the aforementioned exclusion criteria. Differences between reviewers were resolved by discussion. The level of inter-examiner agreement for the first- and second literature-selection stages was expressed by Cohen’s kappa-scores.

### Risk of bias in individual studies

The Cochrane Collaboration’s tool for assessing risk of bias (RoB 2) was used in the case of randomized clinical trials, whereas for nonrandomized studies, the ROBINS-I tool was employed [22].

### Data collection

A data extraction template was generated and based on the study design, patient- and implant-related information, case definition, follow-up period, interventions, comparisons, and primary and secondary outcomes, patient enrollment into supportive therapy following the treatment as well as the study quality.

### Data analyses

Heterogeneity among the studies, meta-analysis (i.e., weighted mean differences (WMDs) and 95% confidence intervals, random effect model to account for potential methodological differences between studies) and forest plots were assessed using a commercially available software program (Comprehensive Meta-Analysis V3, Biostat, Englewood, NJ 07,631 USA). Statistical significance was defined as *p* < 0.05.

## Results

### Search and screening

**Fig. 1:**
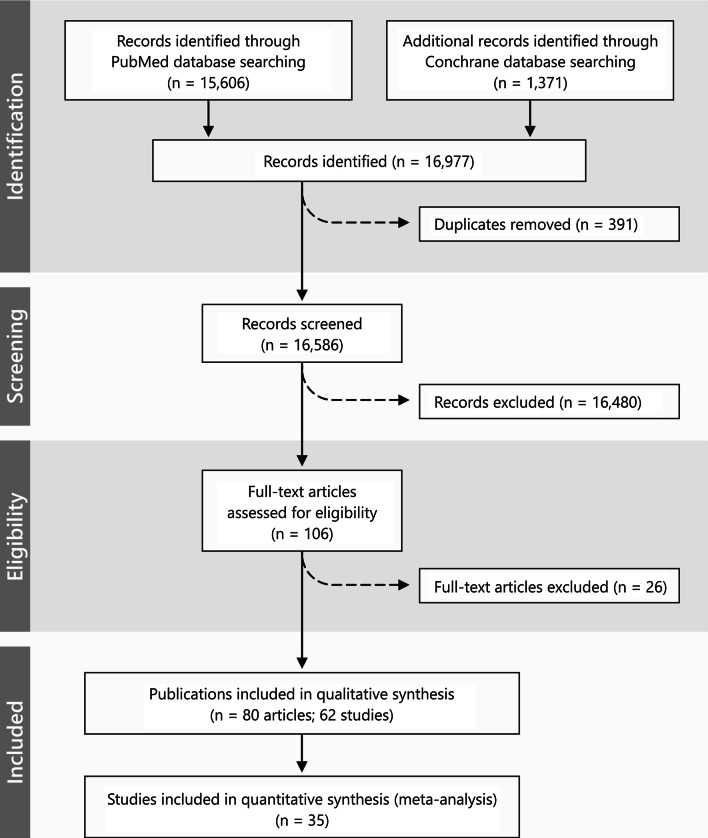
PRISMA flowchart

The screening process yielded 16.586 articles, of which 106 were selected for full-text evaluation (Fig. 1; Cohen’s kappa = 0.723). Upon analysis of the full texts, 26 studies (28 publications) were excluded mainly due to a follow-up period < 6 months (*n* = 8 studies) (for the studies reporting on peri-implantitis treatment) or a lack of a control/comparative treatment group (*n* = 3 studies), or different diagnoses (i.e., peri-implantitis and peri-implant mucositis) being pooled into the analysis (*n* = 2 studies) (Additional file 1). Finally, 80 articles describing 62 studies were included in the review (Cohens kappa = 0.80). Of those studies, 18 reported on the treatment of peri-implant mucositis, 17 reported on non-surgical treatment of peri-implantitis, and the remaining 27 reported on the surgical treatment of peri-implantitis.

### Subdivision of selected studies

All selected studies were subdivided according to differences in the treatment protocol:

Non-surgical treatment of peri-implant mucositis:

- Alternative measures for biofilm removal (4 RCTs [23–26]);
- Adjunctive diode laser/antimicrobial photodynamic therapy (aPDT) (4 RCTs (5 publications) [27–31]);
- Adjunctive local antiseptics (4 RCTs [32–35]);
- Adjunctive systemic antibiotics (2 RCTs [29, 36]);
- Adjunctive probiotics (2 RCTs [37, 38]);
- Adjunctive antiseptic home care mouthrinse (3 RCTs [39–41]).

Non-surgical treatment of peri-implantitis:

- Alternative measures for biofilm removal (5 RCTs (6 publications) [42–47]);
- Adjunctive diode laser/aPDT (2 RCTs [48, 49])
- Adjunctive local antiseptics/antibiotics (6 RCTs (7 publications) [50–56]);
- Adjunctive systemic antibiotics (1 RCT and 1 CCT [57, 58]);
- Adjunctive probiotics (2 RCTs [59, 60]).

Surgical treatment of peri-implantitis:

- Adjunctive and alternative measures for implant surface decontamination following non-reconstructive therapy (7 RCTs (8 publications) [18, 61–67]);
- Adjunctive and alternative measures for implant surface decontamination following reconstructive therapy (1 RCT [68] and 1 CCT [69]);
- Alternative and adjunctive measures for implant surface decontamination following combined therapy (2 RCTs [19, 70]);
- Adjunctive implantoplasty following non-reconstructive therapy (2 RCTs (3 publications) [71–73]);
- Adjunctive local and systemic antibiotics following non-reconstructive therapy (3 RCTs (4 publications) [18, 61, 64, 74]);
- Reconstructive therapy versus non-reconstructive surgery (6 RCTs (7 publications) [75–81]);

Reconstruction of the defect with different bone fillers, with and without a membrane (4 RCTs (5 publications) [82–86] and 3 CCTs (5 publications) [87–91].

### Non-surgical treatment of peri-implant mucositis

The details regarding peri-implant mucositis definitions, non-surgical treatment protocols, and supportive peri-implant therapy are presented in Table 1. The follow-up periods in the included studies were 3 months (9 studies), 4.5 to 8 months (6 studies), and 12 months (3 studies).

**Table 1:** Included studies reporting on peri-implant mucositis treatment

Publication	Design	Population	Case definition	Period	Test	Control	Mean (SD) outcome	Supportive therapy/comments
Alternative measures for biofilm removal								
Ji et al. (2014)	RCT, parallel	24 patients Test: 12; mean age: 46.2 years; 50% female Control: 12; mean age: 41.3 years: 67% female 8 patients – diagnosed with periodontitis 33 implants (test: 17, control: 16) Molar/premolar sites 1 implant system (ITI Straumann, Standard Implant, SLA surface)	PD ≥ 4 mm, BOP + no radiographic bone loss compared with baseline (i.e immediately after prosthesis insertion)	3 months	OHI + mechanical debridement (ultrasonic scaler with carbon fiber tips) + air abrasive device, glycine powder (sites with PD ≥ 4 mm)	OHI + mechanical debridement (ultrasonic scaler with carbon fiber tips)	Subject level BI Test baseline: 1.4 (0.57); 3 months: 1.1 (0.58); * p* = 0.150 Implant level (at sites PD ≥ 4 mm): baseline: 1.7 (0.93); 3 months: 1.1 (0.98); * p* = 0.002 Control subject level: Baseline:1.5 (0.65); 3 months: 1.0 (0.85); * p* = 0.058 Implant level (at sites PD ≥ 4 mm): baseline: 1.7 (1.0); 3 months: 0.9 (1.1); 3-months; * p* < 0.001 Between-group comparison: subject level:* p* = 0.764; implant level: * p* = 0.361 PD Test baseline: 3.6 (0.47) mm; 3 months: 3.2 (0.48); * p* = 0.017 Implant level (at sites PD ≥ 4 mm): baseline: 4.6 (0.50); 3 months: 3.7 (0.95); * p* < 0.001 Control subject level: Baseline: 3.5 (0.50); 3 months: 3.1 (0.38); * p* = 0.012 Implant level (at sites PD ≥ 4 mm): baseline: 4.5 (0.55); 3 months: 3.1 (0.38); 3-months; * p* = 0.012 Between-group comparison: subject level:* p* = 0.587; implant level: * p* = 0.831	During follow-up visits, oral hygiene instructions were reinforced (at 1- and 3-months) Adjunctive air abrasive device with glycine powder appeared to have a limited beneficial effect as compared with mechanical debridement alone
De Siena et al. (2014)	CCT, parallel	30 patients (15 per group) Test: 9 female; 6 male; mean age: 64.8 (12.5) years; mean cigarettes per day: 5.5 (2.6); Control: 9 female, 6 male; mean age: 63.3(9.3) years; mean cigarettes per day: 4.3(2.3); None of the patients were diagnosed with periodontitis	BOP or spontaneous bleeding with local swelling + PD ≤ 3.5 mm + bone loss ≤ 3.0 mm	6 months	OHI + mechanical debridement Teflon curettes, polishing + air abrasive devic with, glycine powder	OHI + mechanical debridement Teflon curettes, polishing	Implant level BI Test: 13 patients did not present bleeding at 6 months; Control: 9 patients did not present bleeding at 6 months PD Test baseline: 3.0 (0.4) mm; 6 months: 2.4 (0.5) mm; * p* < 0.05; Control baseline: 2.9 (0.4) mm; 6 months: 3.0 (0.6) mm; * p* > 0.05	OHI were provided at baseline and repeated in each follow-up visit 3 and 6 months after intervention Test group showed a significant reduction in PD values
Riben-Grundstrim et al. (2015)	RCT, parallel	37 patients Test:17; mean age: 64.4 (range: 25–85) years; Control:18; mean age: 64.3 (range: 25–86) years; 5 patients – current smokers (test: 1; control: 4) 37 implants 3 implant systems (Astra Tech, Nobel Biocare, Straumann)	PD ≥ 4 mm, BOP + with or without SUPP + bone loss ≤ 2 mm from implant shoulder	12 months	OHI + air abrasive device with glycine powder Repeated treatment at 3 and 6 months	OHI + mechanical debridement (ultrasonic scaler with plastic coated tips) Repeated treatment at 3 and 6 months	Subject level BOP Test baseline: 43.9 (7.3)%; 12 months: 12.1 (3.8)%; * p* < 0.05 Control baseline: 53.7 (7.9)%; 12 months: 18.6 (6.4)%; * p* < 0.05 No significant difference between the groups Number of diseased sites (PD ≥ 4 mm with BOP/ SUPP) Test baseline: 38%; 12 months: 8% Control baseline: 52%; 12 months: 17%	Supragingival maintenance care was provided at months 9 and 12 Both treatment approaches were effective in treating peri-implant mucositis
Wohlfahrt et al. (2018)	RCT, split-mouth desing	11 patients Age: NR Periodontal/smoking status: NR 24 implants (test: 12, control: 12) 3 implant brands (Astra, Nobel Mark III, Straumann)	BOP + at least on esite PD ≥ 4 mm + no perceptible bone loss	6 months	Chitosan brush (BioClean Labrida) with oscillating hand piece for 3 min + saline irrigation	Mechanical debridement (titanium curettes) for 3 min + saline irrigation	Implant level mBOP Test baseline: 1.54 (0.78), 6 months: 0.70 (0.70); Control baseline: 1.35 (0.85); 6 months: 0.74 (0.80); no significant difference between groups PD Test baseline: 4.27 (1.36) mm; 6 months: 4.09 (1.68) mm Control baseline: 4.29 (1.50) mm; 6 months: 3.95 (1.27) mm; no significant difference between groups	Reduced signs of inflammation were seen in both groups
*Adjunctive diode laser/aPDT therapy*								
Javed et al. (2017)	RCT, parallel	54 male patients Test: 28 patients, mean age: 50.6 (0.8) years Control: 26 patients, mean age: 52.5 (0.5) years Nr of implants—NR All patients smokers	PD ≥ 4 mm at least at 30% sites	3 months	Mechanical debridement (plastic curettes) + aPDT (photosensitizer: phenothiazine chloride (HELBO)) application for 2 min + light exposure (diode laser 660 nm 10 s.)	Mechanical debridement (plastic curettes)	Subject level BOP Test baseline: 10.2 (1.2)%, 3 months: 8.8 (0.2)%, * p* < 0.001; Control baseline: 8.6 (0.8)%, 3 months: 6.9 (0.2)%, * p* < 0.001. Between group comparison: * p* > 0.001. No significant difference between the groups PD Test baseline: 7.4 (0.3) mm, 3 months: 1.5 (0.3) mm, * p* < 0.001; Control baseline: 6.6 (NR) mm, 3 months: 3.8 (0.4) mm, * p* < 0.001. Between-group comparison: * p* < 0.001	In smokers, aPDT was more effective in the treatment of peri-implant mucositis compared to mechanical debridement alone
Al Rifaiy et al. (2018)	RCT	38 male patients Test: 20 patients; mean age: 33.6(3.8) years; 28 implants Control: 18 patients; mean age: 35.4(2.1) years; 27 implants All patients reported on vaping e-cigarettes	BOP + no bone loss > 2 mm (7^th^ EWON)	3 months	OHI + mechanical debridement + aPDT(photosensitizer: 0.005% Methylene blue) application for 10 s. + diode laser irradiation (670 nm) at 150 milliwatts for 1 min	OHI + mechanical debridement	Subject level BOP Test baseline: 14.6 (3.1)%, 3 months: 11.7 (0.5)%; * p* < 0.001; Control baseline: 9.2 (1.0)%, 3 months: 7.9 (0.2)%; * p* < 0.001. No significant difference between the groups PD Test baseline: 4.3 (0.8) mm; 3 months: 2.1 (0.3) mm; * p* < 0.001; Control baseline:, 4.5 (0.9) mm; 3 months: 2.2 (0.5) mm; * p* < 0.001; Significantly higher reduction in the test group (*p* < 0.001)	aPDT was more effective compared to mechanical debridement alone
Aimetti et al. (2019)	RCT, parallel	220 patients Test: 110 patients; mean age: 58.1(10.1) years; 78 female; light smokers: 14 patients; history of periodontitis: 54 patients Control: 110 patients; mean age: 56.8 (10.2) years; 71 female; light smokers: 20 patients; history of periodontitis: 45 patients 220 implants: 110 test, 110 control	PD ≥ 4 mm + BOP ± SUPP + no radiographic bone loss beyond bone remodeling or (in the absence of baseline radiographic data) < 2 mm	3 months	OHI + diode laser application (980-nm, 2.5 W 30 s. + irrigation with 3% H_2_O_2_ 10 s.(repeated 3 times) + debridement manual and ultrasonic (titanium-coated Gracey or carbon fiber curretes) + biostimulation 60 s. at 0.7 W	OHI + debridement manual and ultrasonic (titanium-coated Gracey or carbon fiber curretes)	Subject level BOP Test baseline: 48.3 (26.9)%, 3 months: 23.3 (23.5), * p* < 0.05 Control baseline: 46.2 (25.6)%, 3 months: 26.8 (23.0)%, * p* < 0.05. Between-group comparison: * p* > 0.05 PD Test baseline: 3.5 (0.7) mm, 3 months: 2.9 (0.6), * p* < 0.05 Control baseline: 3.4 (0.9) mm, 3 months: 3.0 (0.7) mm, * p* < 0.05. Between-group comparison: * p* > 0.05	Reinforcement of OHI 1- and 3-months after the treatment and professional implant cleaning and polishing The adjunctive use of diode laser did not yield any statistical significant clinical benefit as compared to mechanical treatment alone
Mariani et al. 2020 [follow-up study of Aimetti et al. (2019)]		73 patients Tests: 28; mean age: 59.2 (9.3) years; female: 24; history of periodontitis: 12 patients Control: 35; mean age: 62.1(6.8) years; female: 23; history of periodontitis: 13 patients		12 months			Subject level Test baseline: 63.6 (24.2)%, 12 months: 25.8 (24.1); * p* < 0.001 Control baseline: 59.5 (25.0)%, 12 months: 27.6 (25.5)%, * p* < 0.001 Between-group comparison: * p* > 0.05 PD Test baseline: 3.6 (0.7) mm, 12 months: 3.1 (0.7), * p* < 0.001 Control baseline: 3.8 (0.6) mm, 12 months: 3.3 (0.6) mm, * p* < 0.001. Between-group comparison: * p* > 0.05	During recalls (3, 6, 12 months after treatment) OHI reinforcement and professional implant cleaning and polishing The adjunctive use of diode laser showed no statistically significant additional beneficial effect in treatment of peri-implant mucositis
Deeb et al. (2020)	RCT, parallel, 3 arm	45 male patients Test 1: 15 patients; mean age: 52.6(0.9) years; Test 2: 15 patients; mean age: 53.8(0.7) years; Control: 15 patients All patients smokers Nr of implants – NR	BOP + no signs of bone loss	3 months	Test 1 OHI + mechanical debridement with titanium curettes and polishing with rubber cups and paste + aPDT (photosensitizer: Phenothiazine chloride (HELBO) application for 2 min, light exposure (diode laser 660 nm 10 s.) + CHX 0.12% mouthrinse twice daily 2 weeks Test 2 debridement with titanium curettes and polishing with rubber cups and paste + aPDT (aPDT (photosensitizer: Phenothiazine chloride (HELBO) application for 2 min, light exposure (diode laser 660 nm 10 s.) + CHX 0.12% mouthrinse twice daily 2 weeks + Azithromycin 500 mg (1^st^ day), 150 mg (following 2–4 days)	OHI + mechanical debridement with titanium curettes and polishing with rubber cups and paste + CHX 0.12% mouthrinse twice daily 2 weeks	Subject level BOP Test 1 baseline: 12.3 (4.8)%, 3 months: 8.0 (3.7)%; * p* < 0.001 Test 2 baseline: 15.7 (3.9)%, 3 months: 10.1 (3.1)%; * p* < 0.001 Control baseline: 13.6 (4.0)%, 3 months: 11.8 (4.0); * p* < 0.001. Between-group comparison: * p* > 0.05 PD Test 1 baseline: 4.8 (1.0) mm, 3 months: 3.9 (0.9) mm; * p* < 0.001 Test 2 baseline: 4.6 (1.1) mm, 3 months: 3.9 (1.0) mm; * p* < 0.001 Control baseline: 4.5 (0.8) mm, 3 months: 4.1 (1.0); * p* < 0.001. Between-group comparison: * p* > 0.05	Adjunctive aPDT to mechanical debridement was as efficacious as adjunctive antibiotic therapy Additional benefits in reducing BOP scores were observed for adjunctive aPDT among the smokers
*Adjunctive local antiseptic/systemic antibiotic therapy*								
Porras et al. (2002)	RCT, parallel	16 patients Mean age: 58.9 (8.4) years (range: 34–76) Smokers excluded Periodontal status – NR 28 implants Test: 16 Control: 12 3 implant types (plasma-sprayed Ti/ cp Ti (HA-coated Ti)	Supra- and subgingival plaque + PD ≤ 5 mm BOP + “incipient” radiographic lesion	3 months	OHI + mechanical cleansing (plastic scaler, rubber cups, polishing paste) + local irrigation CHX (0.12%) and topical CHX gel application + 0.12% CHX mouthrinse twice for 10 days	OHI + mechanical cleansing (plastic scaler, rubber cups, polishing paste)	Implant level mSBI and BOP (%) scores: no sign. differences between groups at 1 and 3 months PD values Test: baseline: 3.27 (0.81); 3 months: 2.71 (0.70)mm Control: baseline: 3.48 (0.61); 3 months: 2.55 (0.72) mm Changes in mean PD between test and control groups at 3 months were statistically significant (*p* = 0.035)	The addition of CHX to mechanical debridement did not enhance the outcomes as compared to mechanical debridement alone
Thöne-Mühling et al. (2010)	RCT, parallel	11 patients with treated chronic periodontitis Mean age: 51.5 years (range: 37–67) Smokers included 36 implants Tests: 22 Control: 14 2 implant types (MK ii; Nobel Biocare and Osseotite 3i Implant innov.)	BOP + and/or gingival index (GI) ≥ 1 absence of radiographic bone loss during the last 2 years	8 months	OHI + mechanical cleansing (plastic scaler and polyetheretherketone-coated ultrasonic instruments) + topical CHX gel application once + full mouth disinfection (deep scaling in one session + CHX disinfection of tongue and tonsils) + 0.2% CHX mouthrinse 2 × /day and tonsil spraying 1 × /day for 14 days	OHI + mechanical cleansing (plastic scaler and polyetheretherketone-coated ultrasonic instruments) + full mouth scaling in one session	Implant level BOP Test baseline: 0.22 (0.11); 8 months: 0.16 (0.09) % Control baseline: 0.17 (0.19); 8 months: 0.17 (0.11) % PD Test baseline: 3.49 (0.78); 8 months: 2.84 (0.64) mm Control baseline: 3.4 (0.62) mm; 8 months: 2.82 (0.59) mm PD reduced significantly after 8 months compared to baseline (test: * p* = 0.033; control: * p* = 0.004). No significant difference between the groups ML (recession) Test baseline: 0.21 (0.25) mm; 8 months: 0.35 (0.65) mm; Control baseline: 0.33 (0.42) mm; 8 months: 0.33 (0.44) mm	Both treatments lead to an improvements of the clinical parameters, but without significant differences between the groups after 8 months
Hallström et al. (2012)	RCT, parallel	45 patients, 45 implants: 22 test, 23 control Mean age: test 54.6 (18.2) years; control 54.6 (19.8) years	PD ≥ 4 mm BOP + and/or SUPP + radiographic bone loss ≤ 2 mm	6 months	OHI + mechanical cleansing (titanium curettes + rubber cups + polishing paste) + Azithromycin® 500 mg day 1 and 250 mg days 2–4	OHI + mechanical cleansing (titanium curettes + rubber cups + polishing paste)	Subject level BOP Test baseline: 82.6 (24.4)%, 6 months: 27.3 (18.8)% Control baseline: 80.0 (25.0)%, 6 months: 47.5 (32.3)%; Between group comparison: * p* > 0.05 Mean PD Test baseline: 4.4 (1.0) mm, 6 months: 3.5 (1.1) mm Control baseline: 4.1 (0.9) mm; 6 months: 4.1 (1.2) mm; Between-group comparison: * p* < 0.16 Odds ratio of a positive treatment outcome (PD ≤ 4.0 mm and BOP ≤ 1) was 4.5:1 (test vs. control)	No short-term differences were found between study groups
Menez et al. (2016)	RCT, parallel	37 patients: 6 male, 31 female. Age range: 33–75 years, mean age: 57.4 years Only non-smokers included Test: 61 implants; control: 58 implants	BOP + PD ≥ 5 mm + no radiographic evidence of bone loss beyond the first two threads of the implants	6 months	OHI + subgingival debridement with plastic curets + CHX (0.12%) mouthrinse used for brushing the dorsum of the tongue for 1 min. + rinsing for 10 s. + subgingival irrigation for 10 min. + CHX mouthrinse (0.12%) every 12 h, 30 min. 14 days	OHI + subgingival debridement with plastic curets + placebo mouthrinse used for brushing the dorsum of the tongue for 1 min. + rinsing for 10 s. + subgingival irrigation for 10 min. + placebo mouthrinse every 12 h, 30 min. 14 days	Implant level BOP Test baseline: 75.82 (33.98)%, 6 months: 45.76 (34.85)%, * p* < 0.001 Control baseline: 67.54 (34.38)%, 6 months: 41.08 (41.0)%; * p* < 0.001 Between-group comparison: * p* = 0.21 PD Test baseline: 2.85 (0.60) mm; 6 months: 2.49 (0.60) mm; * p* < 0.001; Control baseline: 2.72 (0.68) mm; 6 months: 2.49 (0.67) mm, * p* < 0.001; Between-group comparison: * p* = 0.32	Patients were further motivated with respect to oral hygiene habits during the entire period of the study Use of 0.12% CHX did was not more effective than placebo
Iorio-Siciliano et al. (2020)	RCT, parallel	45 patients Smokers included; Patients with gingivitis or treated periodontitis included; 67 implants Test: 22 patients; mean age: 46.5(15.35) years; 22.7% smokers; 40.9% of patients with treated periodontitis; 33 implants Control: 23 patients; mean age: 45.96(9.84) years; 26.1% smokers; 43.5% of patients with treated periodontitis; 34 implants	≥ 1 site with BOP + absence of radiographic bone loss compared to previous radiograph	6 months	Amino acid buffered sodium hypochlorite (Perisolv) application + ultrasonic scaler + application of Perisolv repeated 5 times + CHX (0.12%) gel twice daily for 2 weeks	Application of placebo gel + ultrasonic scaler + application of placebo gel repeated 5 times + CHX (0.12%) gel twice daily for 2 weeks	Implant level BOP-positive implants Test baseline: 33%, 6 months: 18%; * p* < 0.001 Control baseline: 34%, 6 months: 23%, * p* < 0.001. Between-group comparison: * p* = 0.271 PD Test baseline: 3.93 (1.09) mm, 6 months: 3.04 (0.46) mm; * p* < 0.001; Control baseline: 3.68 (0.85) mm; 6 months: 3.07 (0.58) mm; * p* < 0.001 Between-group comparison: * p* = 0.53	Clinical parameters were recorded at 1, 3 and 6 months following the treatment Test: 110 patients; mean age: 58.1 ± 10.1 years; 78 female; light smokers; 14; history of periodontitis: 54 patients Changes in PD from baseline to 6 months were not statistically significantly different between groups. Complete resolution was not achieved with either of the therapies
*Adjunctive probiotics*								
Pena et al. (2019)	RCT, parallel	50 patients, 50 implants Test: 25 patients; mean age: 55.96(10.81) years; 0% smokers; 64% of patients with a history of treated periodontitis Control: 25 patients; mean age: 61.16(10.62) years; 4% smokers; 68% of patients with a history of treated periodontitis	BOP ± gingival redness, swelling + no bone loss (7^th^ EWOP)	135 days (4.5 months)	OHI + mechanical debridement (ultrasound titanium tip) + CHX 0.12% mouthrine twice a day, 2 weeks + after 15 days: probiotic tablets containing two strains of *L. reunteri* for 1 month	OHI + mechanical debridement (ultrasound titanium tip) + CHX 0.12% mouthrine twice a day, 2 weeks + after 15 days: placebo tablets	Subject level BOP Test baseline: 100%; 135 days: 64% Control baseline: 100%, 135 days: 60%; Between group comparison: * p* = 0.771 PD Test baseline: 3.10 (0.74) mm, 135 days: 2.88 (0.62) mm; Control baseline: 3.32 (0.65) mm, 135 days: 2.98 (0.60) mm; Between-group comparison: * p* = 0.599	None of the patients received any other mechanical periodontal treatment during the follow-up Administration of probiotics did not seem to provide an additional clinical benefit. Complete disease resolution was not always achieved
Galofre et al. (2018)	RCT, parallel	22 patients with history of periodontitis; 22 implants Test: 11 patienst; mean age: 61.5(10.4) years; 27% female Control: 11 patients; mean age: 60.0(9.5) years; 45% female	Inflammed mucosa with BOP and/or suppuration and no evidence of radiographic bone loss (8^th^ EWOP)	3 months	Mechanical debridement (ultrasound with carbon tip and titanium curettes) + 30 probiotic lozenges (once a day, 30 days) (*L. reuteri*, PerioBalance)	Mechanical debridement (ultrasound with carbon tip and titanium curettes) + 30 placebo lozenges (once a day, 30 days)	Subject level BOP Test baseline: 0.61 (0.27)%, 3 months: 0.29 (0.09)%, * p* = 0.01 Control baseline: 0.42 (0.18)%, 3 months: 0.35 (0.22)%, * p* = 0.377 Between-group comparison of the difference baseline-3 months: * p* = 0.024 PD Test baseline:3.84 (0.55) mm, 3 months: 3.35 (0.76) mm, * p* = 0.09 Control baseline: 3.82 (0.64) mm; 3 months: 3.66 (0.62)mm, * p* = 0.187 Between-group comparison: * p* = 0.094	During the study period patients received neither oral hygiene instructions nor any other mechanical periodontal treatment Administration of probiotics together with mechanical debridement improved clinical parameters at peri-implant mucositis sites
*Adjunctive home care mouthrinse*								
Pulcini et al. (2019)	RCT, parallel	46 patients, 54 implants Untreated or recurrent periodontitis patients excluded Test: 24 patients; 27 implants; mean age: 61.3(8.9) years; smokers: 7.4%; 11 female; 6 patients with systemic diseases Control: 22 patients; 27 implants; mean age: 61.0(12.0) years; smokers: 14.8%; 14 female; 4 patients with systemic diseases	BOP and/or SUPP without progressive radiographic bone loss	12 months	OHI + mechanical debridement (ultrasound with plastic tip and air-polishing with erythritol) + home care mouthrinse (0.03% CHX + 0.05% cetylpiridinium chloride (CPC)) (twice a day, 30 s.) 12 months	OHI + mechanical debridement (ultrasound with plastic tip and air-polishing with erythritol) + home care placebo mouthrinse (twice a day, 30 s.) 12 months	Implant level BOP Test baseline: 58.64 (27.49)%, 12 months: 10.42 (13.74)% Control baseline: 46.30 (24.17)%, 12 months: 14.39 (18.04)% Between group comparison: * p* = 0.402 PD Test baseline: 3.36 (0.78) mm; 12 months: 2.50 (0.43) mm Control baseline:3.38 (0.60) mm; 12 months: 2.57 (0.57) mm Between group comparison: * p* = 0.650 After 12 months, 58.3% of test implants, and 50% of control implants demonstrated complete disease resolution (i.e., absence of BOP); * p* > 0.05	The use of test mouthrinse demonstrated some adjunctive benefits. Complete disease resolution could not be achieved in every case
Bunk et al. (2020)	RCT, parallel, three-arm	60 patients/60 implants Test 1: 20 patients, 20 implants Test 2: 20 patients, 20 implants Control: 20 Periodontally healthy Smokers excluded	BOP and/or SUPP + absence of radiographic bone loss compared to previous examination, visual signs of inflammation (modified gingival index ≠ 0)	3 months	Test 1 OHI + mechanical debridement (titanium curettes + polishing with low-abrasive paste) + home use of oral irrigator (Waterpick) with water once a day, 3 months Test 2 OHI + mechanical debridement (titanium curettes + polishing with low-abrasive paste) + home use of oral irrigator (Waterpick) with 0.06% CHX solution once a day, 3 months	OHI + mechanical debridement (titanium curettes + polishing with low-abrasive paste)	Subject level BOP-positive sites Test 1 baseline: 2.25 (1.02), 3 months: 0.45 (0.69) Test 2 baseline: 2.40 (0.88), 3 months: 0.10 (0.45) Control baseline: 2.35 (0.99), 3 months: 0.85 (1.09) Test 2 group showed significantly lower BOP-positive sites compared to control group (*p* = 0.004) After 3 months prevalence of peri-implant mucositis was 5% in Test 2, 35% in Test 1, and 50% in the control group	All patients returned for follow-up and data collection after 4, 8, 12 weeks. Mechanical debridement was not performed at follow-up visits The adjunctive use of oral irrigator with 0.06% CHX in addition to mechanical debridement can reduce the presence of peri-implant mucositis
Philip et al. (2020)	RCT, Parallel 3-arm	Test 1: 31 patients/31 implants; 15 female; mean age: 59 (10.6) years; current smokers: 4; 19 implant in maxilla; 12 in mandible Test 2: 30 patients/30 implants; 14 female; mean age: 62 (9.3) years; current smokers: 2; 19 implant in maxilla; 11 in mandible Control: 28 patients/28 implants; 12 female; mean age: 65 (10.3) years; current smokers: 3; 20 implant in maxilla; 8 in mandible	BOP and/or suppuration with SUPP progressive radiographic bone loss compared to baseline radiograph	3 months	OHI + ultrasonic scaler with plastic tip + homecare mouthrinse: Test 1: Decapinol mouthrinse consisting (0.2%) delmopinol hydrochloride Test 2: CHX (0.2%)	OHI + ultrasonic scaler with plastic tip + homecare Placebo mouthrinse	Subject level mBI Baseline test 1: 1.0 (0.49); 3 months: 0.13 (0.23) Baseline test 2: 1.03 (0.44); 3 months: 0.28 (0.30) Baseline control: 1.08 (0.52); 3 months: 0.19 (0.32); statistically significant reduction compared to baseline (*p* = 0.001); between-group comparison: * p* = 0.42 BOP Baseline test 1: 45 (25.52); 3 months: 3.22 (10.01) Baseline test 2: 43.88 (22.52); 3 months: 8.88 (12.17) Baseline control: 47.02 (24.45); 3 months: 7.73 (13.96); statistically significant reduction compared to baseline (*p* = 0.001); between-group comparison: * p* = 0.14 PD Baseline test 1: 3.18 (0.69) mm; 3 months: 2.65 (0.45) mm Baseline test 2: 3.44 (0.60) mm; 3 months: 2.76 (0.47) mm Baseline control: 3.17 (0.78) mm; 3 months: 2.40 (0.67) mm; statistically significant reduction compared to baseline (*p* = 0.001); between-group comparison: * p* > 0.05 Complete disease resolution (i.e., absence of BOP) 3 months: Test 1: 27 (87%) Test 2: 18 (60%) Control: 20 (71%); *p* = 0.29	Supragingival maintenance care was provided at 1 and 3 months Mechanical debridement combined with OHI is effective in treating peri-implant mucositis

*RCT* randomized clinical trial, *OHI* oral hygiene instructions, *BOP* bleeding on probing, *mBOP* modified bleeding on probing index, *PD* probing depth, *SUPP* suppuration, *BI* bleeding index, *mBI* modified bleeding index, *aPDT* antibacterial photodynamic therapy

Marked inconsistencies in case definitions for peri-implant mucositis appeared among the studies. Specifically, in all but 1 study [27], peri-implant mucositis diagnosis was based on the presence of BOP and/or SUPP, along with a radiographic MBL assessment. Regarding an MBL assessment, a peri-implant mucositis diagnosis was defined via an absence of bone loss compared to the baseline radiograph or via threshold values (i.e., ≤ 3 mm or ≤ 2 mm). In 9 studies, peri-implant mucositis diagnosis was supplemented by an assessment of PDs, with the large variations in the applied threshold values.

Three RCTs reported on patients’ enrollment into a supportive maintenance program [25, 30, 31, 41]. All treatments implemented for peri-implant mucositis resulted in improved clinical parameters. However, complete disease resolution (i.e., absence of BOP) rarely occurred throughout the short investigation periods (Table 1).

### Efficacy of interventions

#### Alternative measures for biofilm removal

Alternative measures utilized to remove biofilm from contaminated implant surfaces (i.e., air-powder abrasive devices with glycine powder or chitosan brush) showed no beneficial clinical effect in terms of BI/BOP and PD values compared to the control treatment alone (i.e., mechanical debridement) [23–26].

#### Adjunctive diode laser/aPDT

In 4 RCTs (5 publications), either antimicrobial photodynamic therapy (aPDT) [27–29] or a diode laser [30, 31] was used in addition to mechanical debridement. Over a 3-month period, adjunctive use of aPDT led to similar treatment outcomes in terms of BOP [27–29] and PD changes [28, 29], while 1 study reported on a higher reduction in PD values for the sites treated with adjunctive aPDT [27]. Similarly, the additional application of a diode laser resulted in similar BOP and PD changes compared to the mechanical treatment alone over 3- and 12-month periods [30, 31].

#### Adjunctive local antiseptics

As an adjunct to mechanical debridement, included studies employed either applications of CHX (0.12%) gel [32], a full-mouth disinfection concept utilizing CHX gel and mouth rinse [33, 34], or applications of sodium hypochlorite [35]. Over a 3- to 6-month follow-up period, adjunctive use of the aforementioned local antimicrobials led to similar changes in BOP scores [32, 34, 35] and PD values [33–35] compared to control treatments (i.e., mechanical debridement alone), whereas one study reported on a greater PD reduction following the adjunctive use of local CHX (0.12%) applications [32].

#### Adjunctive systemic antibiotics

The potential beneficial effect of adjunctive systemic antibiotic use for peri-implant mucositis treatment was investigated in 2 RCTs [29, 36]. In particular, administration of systemic antibiotics (azithromycin) along with mechanical debridement [36] or in combined with subgingival debridement and aPDT therapy [29] failed to show any beneficial effect upon the changes of BOP and PD values over follow-up periods of 3- to 6-months.

#### Adjunctive probiotics

Two RCTs investigated the potential benefits of probiotics [37, 38]. Of those, 1 RCT in which probiotics were administered for 15 days following the mechanical treatment failed to detect additional beneficial effects of probiotics in BOP and PD changes compared to the controls [37]. Another RCT pointed to significantly higher BOP reduction following the adjunctive use of probiotics for 30 days compared to the controls, whereas changes in PD values were similar to those obtained in the control group [38].

#### Adjunctive antiseptic home care mouth rinse

Three RCTs investigated the possible beneficial effect of home care use of cetylpiridinum chloride (CPC) + CHX 0.03% mouth rinse [40], oral irrigator with or without 0.06% CHX [39], or CHX 0.2% mouth rinse compared to 0.2% delmopinol hydrochloride [41]. Although 2 of them found similar BOP and PD changes irrespective of the adjunctive use of home care antibacterial mouth rinse throughout a 3-month follow-up period [40, 41], the remaining RCT indicated significantly higher BOP reduction for the patients in the test group [39].

### Synthesis of results

#### Alternative measures for biofilm removal

**Fig. 2:**
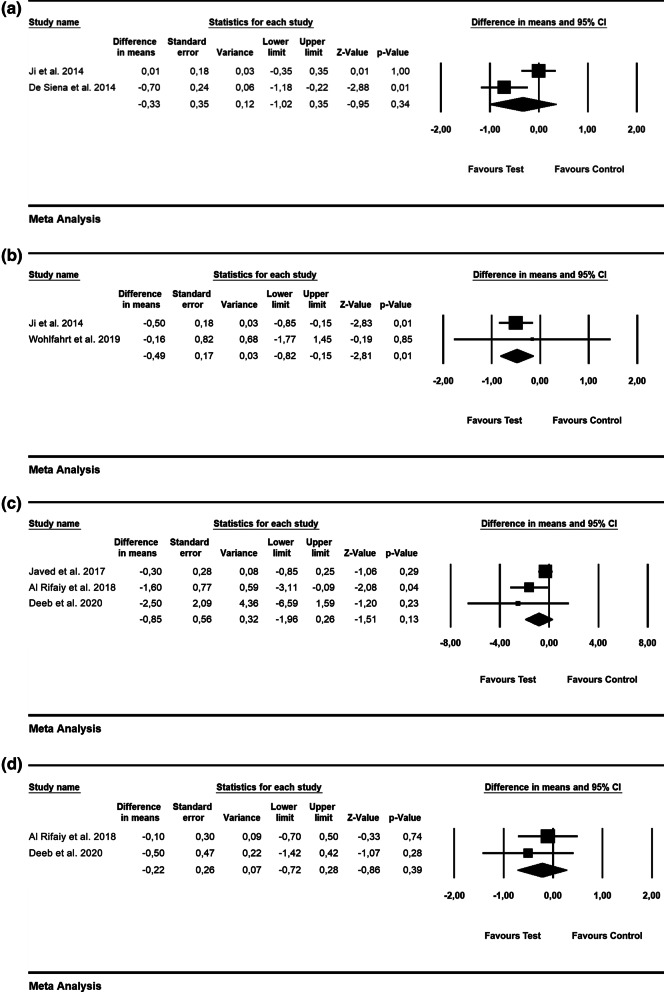

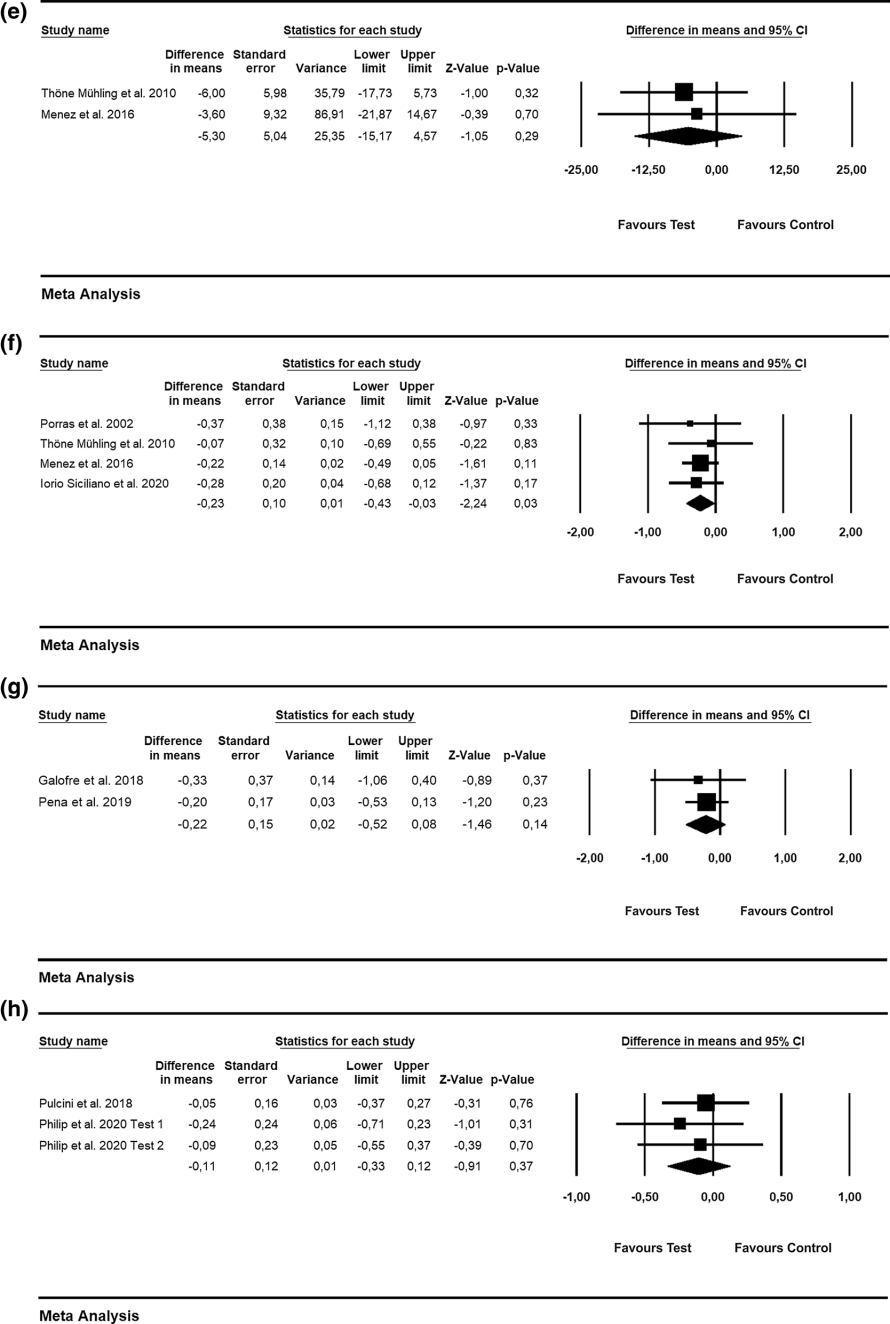
Forest plots indicating weighted mean difference (95% CI) in the changes of the assessed treatment outcomes following non-surgical treatment of peri-implant mucositis. **a** Alternative measures for biofilm removal (patient-level analysis)—PD. **b** Alternative measures for biofilm removal (implant-level analysis)—PD. **c** Adjunctive aPDT (patient-level analysis)—BOP. **d** Adjunctive aPDT (patient-level analysis)—PD. **e** Adjunctive local antiseptic therapy (implant-level analysis)—BOP. **f** Adjunctive local antiseptic therapy (implant-level analysis)—PD. **g** Adjunctive probiotics (implant-level analysis)—PD. **h** Adjunctive home care mouthrinse (implant-level analysis)—PD

Based on the patient-level analysis, the WMD in PD values were − 0.33 mm [SE = 0.35; *p* = 0.34; 95% CI (− 1.02, 0.35)], not favoring the use of alternative measures (i.e., air powder abrasive device with glycine powder) for biofilm removal (p value for heterogeneity: 0.02, I^2^ = 81.5% = substantial heterogeneity) [23, 24] (Fig. 2a). At the implant level, WMD in PD amounted to − 0.49 mm [SE = 0.17; *p* = 0.01; 95% CI (− 0.82, − 0.15)], thus pointing to no favorable effect of alternative measures (i.e., air abrasive device with glycine powder and chitosan brush) for biofilm removal compared to mechanical debridement (p value for heterogeneity: 0.00, I^2^ = 0.0% = low heterogeneity) [23, 26] (Fig. 2b).

#### Adjunctive aPDT

The WMD in BOP and PD values were − 0.85% [SE = 0.56; *p* = 0.13; 95% CI (− 1.96, 0.26)] and − 0.22 mm [SE = 0.26; *p* = 0.39; 95% CI (− 0.72, 0.28); unit of analysis: patient], respectively, thus not favoring the adjunctive use of aPDT compared to mechanical debridement alone (p value for heterogeneity: 0.013, I^2^ = 77% = substantial heterogeneity and *p* = 0.747, I^2^ = 0.0% = low heterogeneity, repsectively) (Fig. 2c and d) [27–29].

#### Adjunctive local antiseptics

The WMD in BOP amounted to − 5.30% [SE = 5.04; *p* = 0.29; 95% CI (− 15.06, 4.57); unit of analysis: implant], thus not supporting the superiority adjunctive use of local antiseptics (i.e., CHX) along with mechanical debridement (2 RCTs; p value for heterogeneity: 0.828, I^2^ = 0.0% = low heterogeneity; Fig. 2e) [33, 34]. Based on 4 RCTs, the WMD in PD values was − 0.23 mm [SE = 0.10; *p* = 0.03; 95% CI (− 0.43, − 0.03); unit of analysis: implant], favoring the adjunctive use of local antiseptics (i.e., CHX and sodium hypochlorite; p value for heterogeneity: 0.929, I^2^ = 0.0% = low heterogeneity; Fig. 2f) [32–35].

#### Adjunctive probiotics

According to 2 RCTs, the WMD in PD values amounted to − 0.22 mm [SE = 0.15; *p* = 0.14; 95% CI (− 0.52, 0.08); unit of analysis: implant], suggesting no superiority of probiotics in terms of PD reduction (p value for heterogeneity: 0.749, I^2^ = 0.0% = low heterogeneity; Fig. 2g) [37, 38].

#### Adjunctive antiseptic home care mouthrinse

Based on 2 RCTs, the estimated WMD in PD amounted to − 0.11 mm [SE = 0.12; *p* = 0.37; 95% CI (− 0.33, 0.12); unit of analysis: implant), not favoring the use of adjunctive antiseptic home care mouthrinse as an adjunct to mechanical debridement (*p* value for heterogeneity: 0.8, *I*^2^ = 0.0% = low heterogeneity; Fig. 2h) [40, 41].

### Non-surgical treatment of peri-implantitis

Peri-implantitis definitions, non-surgical treatment protocols, and supportive peri-implant therapies are addressed in Table 2. The follow-up periods in the included studies were either 6 months (10 studies) or 12 months (7 studies).

**Table 2:** Included studies reporting on non-surgical treatment of peri-implantitis

Publication	Design	Population	Case definition	Period	Test	Control	Mean (SD) outcome	Supportive therapy/comments
*Alternative measures for biofilm removal*								
Schwarz et al. (2005)	RCT, parallel	20 patients Test: 10, mean age: 48 years Control: 10, mean age: 51 years Smokers excluded 32 implants rough and medium-rough surfaces	PD ≥ 4 mm + BOP/SUPP + radiographic bone loss	6 months	OHI + Er:YAG laser device (cone-shaped glass fiber tip) at 12.7 J/cm2	OHI + mechanical debridement (plastic curettes), 0.2% CHX pocket irrigation and 0.2% CHX gel	Subject level BOP Test baseline: 83.2 (17.2)%, 6 months: 31.1 (10.1)%; * p* < 0.001 Control baseline: 81.3 (19.0)%, 6 months: 58.3 (16.9)%; * p* < 0.001 Between group comparison: * p* < 0.001 PD Test baseline: 5.4 (1.2) mm, 6 months: to 4.6 (1.1) mm; * p* < 0.001 Control baseline: 5.5 (1.5) mm, 6 months: 4.8 (1.4) mm; * p* < 0.001 Between group comparison: * p* > 0.05 ML (recession) Test baseline: 0.4 (0.6) mm, 6 months: 0.5 (0.6) mm Control baseline: 0.7 (0.8)mm, 6 months: 0.8 (0.8) mm	Er:YAG treatment results in statistically significantly higher reduction of BOP
Schwarz et al. (2006)	RCT, parallel	18 patients Test: 10; mean age: 56 (14) years Control: 8; mean age: 54 (11) years Smokers excluded 36 implants rough and medium-rough surfaces	PD ≥ 4 mm + BOP/SUPP + radiographic bone loss	12 months	OHI + Er:YAG laser device (cone-shaped glass fiber tip) at 12.7 J/cm2	OHI + mechanical debridement (plastic curettes), 0.2% CHX pocket irrigation and 0.2% CHX gel	Subject level Moderately deep sites (PD 4–6 mm) BOP Test baseline: 81.7 (6.7)%, 12 months: 35.0 (6.3) %; * p* < 0.01 Control baseline: 81.6 (5.2)%, 12 months: 53.3 (7.3) % Deep sites Test baseline: 79.9 (4.8)%, 12 months: 55.0 (6.5)% Control baseline: 88.3 (3.5)%, 12 months: 66.6 (5.5) % Significantly higher reduction in test group (*p* < 0.01) PD (PD > 7 mm) Moderately deep sites Test baseline: 4.5 (1.4) mm, 12 months: 4.0 (0.1) mm Control baseline: 4.4 (0.2) mm, 12 months: 4.3 (0.1) mm Deep sites Test baseline: 5.9 (0.1) mm, 12 months: 5.4 (0.1) mm Control baseline: 5.9 (0.3) mm. 12 months: 5.5 (0.2) mm No significant differences between groups	Er:YAG treatment results in significantly higher BOP reduction, however, its effectiveness seemed to be limited to a period of 6 months
Renvert et al. (2009)	RCT, parallel	31 patients Test: 14; mean age: 60.3(12.9) years; 7 female; 7 male; 2 patients current smokers Control: 17; mean age: 62.7(12.1) years; 7 female; 10 male; 3 current smokers; 31 implants machined and medium-rough surfaces	PD ≥ 4 mm + BOP/SUPP + bone loss < 2.5 mm	6 months	OHI + ultrasonic device with hydroxyapatite fluid polish	OHI + mechanical debridement (titanium curettes)	Subject level BI Test baseline: 1.7 (0.6); 6 months: 1.2 (0.7) Control baseline: 1.7 (0.9); 6 months: 1.4 (1.0) No significant differences between groups PD Test baseline: 4.3 (0.6) mm, 6 months: 3.9 (0.8) mm Control baseline: 6.2 (1.6) mm; 6 months: 6.3 (2.2) mm No significant differences between groups	All patients received OHI at all study timepoints No group differences were found in the treatment outcomes
Renvert et al. (2011)	RCT, parallel	42 patients Tests: 21; mean age: 68.5 (6.4) years; Control: 21; mean age: 68.9 (12.5) years Smokers included 90 implants machined and medium-rough surfaces	PD ≥ 5 mm, BOP + and/or SUPP + bone loss > 3 mm	6 months	OHI + air abrasive device with glycine powder	OHI + Er:YAG laser device (cone-shaped glass fiber tip, 12.7 J/cm2)	Implant level BOP 6 months Test: 25% of implant showed no bleeding Control: 30.9% of implant showed no bleeding Between-group comparison:* p* = 0.22 PD change Tests: 0.9 (0.8) mm Control: 0.8 (0.5) mm Between-group comparison:* p* = 0.55 RBL change: Test: − 0.3 (0.9)mm Control: − 0.1 (0.8)mm No significant differences between groups Positive treatment outcome (i.e., PD reduction ≥ 0.5 mm + no further bone loss): Test: 47% Positive treatment outcome: Control: 44% Between-group comparison:* p* = 0.84	All patients received OHI at all study timepoints The clinical treatment results were limited and similar between the two methods compared with those in cases with severe peri-implantitis
Sahm et al. 2011, John et al. (2011)	RCT, parallel	30 patients Smokers excluded Test: 15 patients, 22 implants Control: 15 patients, 19 implants 8 implant systems 12 months 25 patients Test: 12 patients Control: 13 patients	PD ≥ 4 mm + BOP with SUPP + bone loss ≤ 33%	12 months	OHI + air abrasive device with glycine powder	OHI + mechanical debridement (carbon curettes + 0.1% CHX)	Subject level BOP Test baseline: 99.0 (4.1)%, 12 months: 57.8 (30.7) % Control baseline: 94.7 (13.7)%, 12 months: 78.1 (30.0) %; Between-group comparison: * p* < 0.05 PD Test baseline: 3.7 (1.0) mm, 12 months: 3.2 (1.1)mm Control baseline: 3.9 (1.1) mm, 12 months: 3.5 (1.2) mm Between-group comparison: * p* > 0.05 ML (recession) Test baseline: 1.5 (1.4) mm, 12 months: 0.1 (0.9) mm Control baseline: 1.0 (1.1) mm, 12 months: 0.1 (0.7) mm; Between-group comparison: * p* > 0.05	Supragingival professional implant/tooth cleansing and reinforcement of oral hygiene was performed at each follow-up visit: 2,4,6,8,10,12 and 24 weeks after the treatment Both treatment procedures results in comparable outcomes
*Adjunctive diode laser/aPDT therapy*								
Arisan et al. (2015)	CCT, parallel	10 patients Patients with ongoing or history of periodontitis excluded 48 two piece, rough-surface implants	BOP + pain ± SUPP + PD 4–6 mm + marginal bone loss < 3 mm	6 months	Suprastructures removed + mechanical debridement with plastic curette + diode laser (810 nm) 1 min + irrigation with sterile saline solution	Suprastructures removed + mechanical debridement with plastic curette + irrigation with sterile saline solution	Implant level BOP Test baseline: 100%; 6 months: 95.8% Control baseline: 100%, 6 months:100% PD Test baseline: 4.71 (0.67) mm, 6 months: 4.54 (0.74) mm Control baseline: 4.38 (0.42) mm, 6 months: 4.17 (0.41) mm RBL Test baseline: 2.12 (0.47) mm, 6 months: 2.79 (0.48) mm Control baseline: 2.35 (0.56), 6 months: 2.63 (0.53) mm No statistically significant difference between the groups	Adjunctive use of diode laser did not yield any additional positive influence compared with conventional scaling
Wang et al. (2019)	RCT, parallel	131 patients Patients with long history of smoking excluded Test: 66; mean age: 42.6 (13.0) years; patients with history of smoking: 21 Control: 65; mean age: 42; mean age: 44.1(9.8) years; patients with history of smoking: 13	PD ≥ 6 mm + BOP + radiographic bone loss	6 months	OHI/full mouth cleansing 2 weeks prior to the therapy + subgingival air abrasive device with glycine powder + irrigation with sterile saline + aPDT (toluidine blue photosensitized application for 3 min.) + 19 s. light emitting didoe lidht (LED)	OHI/full mouth cleansing 2 weeks prior to the therapy + subgingival air abrasive device with glycine powder + irrigation with sterile saline	Subject level SBI Test baseline: patients with no bleeding 0%, 6 months: 93.8% Grade 1 Control baseline: patients with no bleeding 0%, 6 months: 81.8% Grade 3; Significantly higher reduction in the test group (*p* < 0.001) PD Test baseline: 4.93 (1.07); 6 months: 3.06 (0.29), * p* < 0.001 Control baseline: 5.07 (0.72), 6 months: 4.62 (0.45), * p* < 0.001. Significantly higher reduction in the test group (*p* < 0.001)	Adjunctive aPDT significantly improved PD and SBI values
*Adjunctive local antiseptic/antibiotic therapy*								
Renvert et al. (2006)	RCT, parallel	32 patients Test: 16; mean age: 65.5 (8.6) years; female: 7; male: 9; present smokers: 5; former smokers: 6 Control: 14; mean age: 61.1 (8.6) years; 11 female; 3 male; present smokers: 3; former smokers: 7 1–5 (test)/1–6 (control) implants per patient machined surfaces	PD ≥ 4 mm + BOP + SUPP + bone loss ≤ 3 threads	12 months	OHI + mechanical debridement (scalers + rubber cup + polishing) + 1 mg minocycline microspheres	OHI + mechanical debridement (scalers + rubber cup + polishing) + 1.0% CHX gel	Subject level BOP Test baseline: 88 (12)%, 12 months: 71 (22) % Control baseline: 86 (14)%, 12 months: 78 (13) % No significant difference between the groups PD Test baseline: 3.9 (0.7) mm, 12 months: 3.6 (0.6) mm Control baseline: 3.9 (0.3) mm, 12 months: 3.9 (0.4) mm Significantly higher reduction in the test group (*p* < 0.001)	Use of local antibiotics as an adjunct to mechanical treatment og incipient peri-implantitis lesions demonstrated improvements in PD values that were sustained over 12 months
Renvert et al. (2008)	RCT, parallel	32 patients Test: 17; mean age: 60.82 (12.72) years; female: 13, male: 4; present smokers: 2; former smokers: 8 Control: 15; mean age: 62.40 (7.72) years; 9 female, 6 male; present smokers: 5; former smokers: 3 95 implants machined surfaces	PD ≥ 4 mm + BOP + SUPP + bone loss ≤ 3 threads	12 months	OHI + mechanical debridement + 1 mg minocycline microspheres Treatment was repeated at days 30 and 90	OHI + mechanical debridement + 0.5 ml of 1.0% CHXgel Treatment was repeated at days 30 and 90	Implant level BOP Test baseline: 86.5 (20.1)%, 12 months: 48.1 (20.7) %; * p* < 0.001 Control baseline: 89.2 (17.2)%, 12 months: 63.5 (19.2) %; * p* < 0.001 Significantly higher reduction in test group PD Test baseline: 3.85 (1.04) mm, 12 months: 3.55 (0.98) mm; * p* < 0.001 Control baseline: 3.87 (1.16) mm, 12 months: to 3.72 (1.02) mm; * p* < 0.001 No significant difference between the groups RBL Test baseline: 0.77 (0.85) Mm, 12 months: 0.7 (0.85)mm Control baseline: 0.41 (0.7) mm, 12 months: 0.46 (0.76) mm No significant difference between groups	The use of repeated local antibiotics as an adjunct to mechanical treatment of peri-implantitis lesions demonstrated significant improvements in BOP values
Schär et al. 2012, Bassetti et al. (2013)	RCT, parallel	40 patients Test: 20; 10 female; mean age: 59 (range: 27–78); 18 patients with history of periodontitis Control: 20; 10 female; mean age: 57 (range: 29–75) Smokers excluded 40 implants medium rough surfaces	PD 4–6 mm + BOP + bone loss = 0.5–2 mm	12 months	OHI + mechanical debridement (titanium curettes + glycine powder air polishing, pocket irrigation using 3% H_2_O_2_) + aPDT (660 nm, phenothiazine chloride dye)	OHI + mechanical debridement (titanium curettes + glycine powder air polishing, pocket irrigation using 3% H_2_O_2_) + minocycline microsphere	Subject level BOP change Test: 57% Control: 65% PD change Test: 0.56 mm Control: 0.11 mm Complete resolution of mucosal inflammation Test: 31.6% Control: 35% No significant differences between groups	Oral hygiene reinforcement at 1,2,3,8 weeks. In the presence of BOP at implant sites after 3 and 6 months, additional treatment procedures equivalent to initial therapy was provided Mechanical debridement with aPDT was equally effective in reducing mucosal inflammation as with adjunctive delivery of local antibiotics
Machtei et al. (2012)	Multicentre RCT, parallel	60 patients 77 implants Test: 30 patients; 40 implants; mean age: 57.42 (10.5) years; 20 female; current smokers: 5, former smokers: 7 Control: 30 patients; 37 implants; mean age: 60.95(7.9) years; 15 female; current smokers: 5, former smokers: 6	PD 6–10 mm + BOP + radiographic bone loss	6 months	OHI + ultrasonic debridement + matrix containing 2.5-mg CHX chips (i.e., up to 4 per implant site) Repeated application at sites with PD ≥ 6 mm at 2, 4, 6, 8, 12 and 18 weeks	OHI + ultrasonic debridement + biodegradable crosslinked gelatin matrix chip Repeated application at sites with PD ≥ 6 mm at 2, 4, 6, 8, 12 and 18 weeks	Subject level BOP Test baseline: 100 (0.0)%, 6 months: 42.5 (50.0) % Control baseline: 100 (0.0)%, 6 months: 54.5 (50.5) % PD Test baseline: 7.6 (1.1), 6 months: 5.47 (1.86)mm Control baseline: 7.21 (1.08) mm, 6 months: 5.48 (1.25) mm BOP and PD reductions not significantly different between groups	At 12 weeks, supragingival debridement was performed Treatment in both groups results in a substantial improvement
Machtei et al. (2020)	Multicentre RCT, parallel	290 patients Test: 146; mean age: 62.5 (11.2) years; female: 91; current smokers: 15; former smokers: 51 Control: 144; mean age: 62.6 (11.6) years; female: 81; current smokers: 14; former smokers: 55 386 implants Test: 197 Control: 189 10 centers	PD 5–8 mm + BOP/SUPP + radiographic bone loss at least 3 mm from implant shoulder	6 months	OHI + subgingival debridement at baseline and 3 months + matrix containing 2.5-mg CHX chips (i.e., up to 2 per implant site) Repeated supragingival plaque removal and CHX chips application for 12 weeks every 2^nd^ week	OHI + subgingival debridement at baseline and 3 months Repeated supragingival plaque removal for 12 weeks every 2nd week	Subject level BOP Test baseline: 100%, 6 months: 50.31% Control baseline: 100%, 6 months: 55.21% PD Test baseline: 6.16 (1.0) mm, 6 months: 4.40 (1.25) mm Control baseline: 6.06 (0.92), 6 months: 4.52 (1.27) mm. Significantly higher reduction in test group (*p* = 0.01) ML (recession) Test baseline: 0.51 (0.99) mm, 6 months: 0.80 (1.21)mm Control baseline: 0.26 (0.72), 6 months: 0.42 (0.85) mm. Significantly higher change in test group (*p* = 0.0017)	Test group showed significantly greater improvements in PD values
Merli et al. (2020)	RCT, parallel	58 patients Patients with intreated periodontitis excluded 58 implants Test 1 (adjunctive desiccant material): 15; mean age: 60.3(10.7) years; female: 12; smokers: 4 Test 2 (adjunctive air-flow): 13; mean age: 66.4(9.4) years; 9 female; 2 smokers Test 3 (air-flow + desiccant material): 14; mean age: 60.3(8.5) years; 10 female; 4 smokers Control: 16; mean age: 64.5(8.3) years; smokers: 3; female: 9	PD 5–8 mm + BOP/SUPP + bone loss beyond initial bone remodeling + infraosseous defect component (radiographic) ≤ 5 mm + radiographic suprabobe component of defect ≤ 4 mm	6 months	OHI + supragingival/supramucosal debridement prior to treatment Removal of prosthetic reconstruction + mechanical debridement with ultrasonic scaler + Test 1: application of desiccant material (gel of concentrated mixture of hydroxybenzenesulphonic and hydromethyloxybenzene acids and sulphuric acid (HybenX)) in peri-implant pocket for 30 s. followed by saline rinsing Test 2: + air polishing with glycine powder Test 3: Test 2 + Test 1 + 0.12% CHX mouthrinse twice a day, 15 days	OHI + supragingival/supramucosal debridement prior to treatment Removal of prosthetic reconstruction + mechanical debridement with ultrasonic scaler	Subject level BOP (number of sites per implant with bleeding) Test 1 baseline: 2.9 (1.3); 6 months: 2.5 (1.7) Test 2 baseline: 3.6 (0.8); 6 months: 2.8 (1.3) Test 3 baseline: 3.6 (0.8); 6 months: 2.7 (1.3) Control baseline: 3.3 (0.8); 6 months: 2.9 (0.8) No significant difference between groups PD Test 1 baseline: 5.0 (1.2) mm; 6 months: 4.5 (1.2) mm Test 2 baseline: 5.1 (1.5) mm; 6 months: 4.8 (1.3) mm Test 3 baseline: 4.9 (1.1) mm; 6 months: 4.0 (1.2) mm Control baseline: 4.4 (1.1) mm; 6 months: 4.2 (1.3) mm Reduction was higher in patients treated with desiccant material ML (recession) Test 1 baseline: 0.4 (0.5) mm; 6 months: 0.3 (0.5) mm Test 2 baseline: 0.2 (0.9) mm; 6 months: 0.3 (0.7) mm Test 3 baseline: 0.1 (0.2) mm; 6 months: 0.2 (0.4) mm Control baseline: 0.1 (0.1) mm; 6 months: 0.1 (0.2) mm No significant difference between groups Radiographic mean bone defect Test 1 baseline: 3.9 (1.2) mm; 6 months: 4.0 (1.8) mm Test 2 baseline: 3.6 (1.7) mm; 6 months: 4.0 (1.8) mm Test 3 baseline: 3.6 (1.7) mm; 6 months: 3.5 (1.0) mm Control baseline: 3.3 (1.2) mm; 6 months: 3.1 (1.5) mm No significant difference between groups Treatment success (no PD ≥ 5 mm with BOP/SUPP + no further bone loss): Test 1: 3 (25%) Test 2: 2 (14%) Test 3: 6 (43%) Control: 6 (37%) No significant difference between groups	Patients were seen at 1 wee, 1 month, 3 and 6 months for maintenance with supragingival prophylaxis Pocket reduction was more pronounced in groups treated with adjunctive desiccant material
**Adjunctive systemic antibiotics**								
Gomi et al. (2015)	RCT, parallel	20 patients; 11 female; mean age: 67.6 (5.3) years (range: 55–78) All patients periodontally compromised Smokers excluded	PD > 5 mm + BOP + bone loss > 2 mm	12 months	Azithromycin® 500 mg 3 days before treatment + mechanical full-mouth scaling (implants with plastic curettes and plastic ultrasonic scaler)	Mechanical full-mouth scaling (implants with plastic curettes and plastic ultrasonic scaler)	Subject level BOP Test baseline: 27.9 (4.3)%, 6 months: 4.4 (0.3)% Control baseline: 25.7 (4.3)%, 6 months: 19.8 (5.7) %; Between-group comparison: * p* < 0.001 PD Test baseline: 4.28 (0.85) mm, 6 months: 4.35 (0.22) mm Control baseline: 5.7 (0.8) mm; 6 months: 4.22 (0.29) mm; Between-group comparison: * p* = 0.002	All clinical parameters showed better improvements in test group
Shibli et al. (2019)	CCT, parallel	40 patients; 29 female; mean age: 58.5 (11.1) years Current smokers excluded	PD > 5 mm + bone loss > 4 mm + BOP	12 months	Mechanical debridement with teflon curettes + metronidazole 400 mg and amoxicillin 500 mg three times a day, 14 days	Mechanical debridement with teflon curettes	Subject level BOP Test baseline: 90.0 (31.6)%, 12 months: 39.0 (48.8%), * p* < 0.05 Control baseline: 97.0 (34.5)%, 12 months: 50.0 (53.5%); * p* < 0.05; Between-group comparison: * p* > 0.05 PD Test baseline: 9.9 (2.6)mm, 12 month: 5.1 (1.8), * p* < 0.05 Control baseline: 7.6 (1.8) mm, 12 months: 3.8 (1.6), * p* < 0.05; Between-group comparison: p > 0.05	Periodontal supragingival maintenance therapy every 3 months Addition of systemic antibiotics to the mechanical treatment of severe peri-implantitis did not improve clinical outcomes
*Adjunctive probiotics*								
Tada et al. (2017)	RCT, parallel	30 patients Test: 15; mean age: 68.80 (7.46) years; 3 smokers Control: 15; mean age: 65.87 (8.84) years; 1 smokers	PD > 4 mm and < 7 mm + BOP/SUPP + bone loss > 2 mm	6 months	OHI + supragingival scaling + Azithromycin® 500 mg once a day for 3 days + after 1 week probiotic tablets (L. reuteri two strains; ProDentis) once a day for 6 months	OHI + supragingival scaling + Azithromycin® 500 mg once a day for 3 days	Subject level mBI Test baseline: 3.20 (1.26), 6 months: 1.53 (1.41), * p* = 0.235 Control baseline: 3.67 (1.59), 6 months: 2.33 (1.95), * p* = 0.375 PD Test baseline: 3.90 (0.60), 6 months: 3.21 (0.84), * p* = 0.033 Control baseline: 4.04 (1.14) mm, 6 months: 3.47 (0.95) mm, * p* = 1	PDs were significantly reduced only in test group
Laleman et al. (2019)	RCT, parallel	19 patients Smokers and patients with uncontrolled periodontitis excluded Test: 9; 4 female; mean age: 64 (11) Control: 10; 6 female; mean age: 69 89)	PD ≥ 4 mm + BOP + bone loss at least 1 mm compared to the baseline	6 months	OHI + mechanical debridement (titanium curettes + scaler) + Air polishing + topical application of probiotic drops containing L. reuteri two stains + probiotic tablets (BioGaia) for 6 months	OHI + mechanical debridement (titanium curettes + scaler) + Air polishing + topical application of placebo drops + placebo tablets for 6 months	Subject level BOP (number of bleeding sites pro implants 0 to 6) Test baseline: 87 (23)%, 6 months: 59 (32), * p* < 0.01 Control baseline: 87 (22), 6 months: 53 (39), * p* < 0.01 Between-group comparison: * p* = 0.876 PD Test baseline: 5.17 (0.92), 6 months: 4.15 (0.96), * p* < 0.01 Control baseline: 5.45 (1.20), 6 moths: 4.18 (1.26), * p* < 0.01 Between-group comparison: * p* = 0.801	No adjunctive effects of the use of L. reuteri probiotics were found

*RCT* randomized clinical trial, *CHX* chlorhexidine digluconate, *OHI* oral hygiene instructions, *BOP* bleeding on probing, *PD* probing depth, *SUPP* suppuration, *BI* bleeding index, *mBI* modified bleeding index, *RBL* radiographic bone level, *RDF* radiographic bone defect fill, *ML* soft-tissue level, *H*_*2*_*O*_*2*_ hydrogene peroxide, *aPDT* antibacterial photodynamic therapy

Definitions of peri-implantitis varied widely among the included studies. All studies defined peri-implantitis as the presence of BOP and/or SUPP and radiographic MBL. The reference points (i.e., baseline radiographs) and threshold values used to identify MBL were either not specified [42, 43, 48, 54] or exhibited large variations [44–47, 49–53, 55–60].

In four RCT’s patients were enrolled into a regular maintenance program following the treatment [44–47, 58]. Although the investigated clinical parameters tended to improve significantly 6 to 12 months after the implemented non-surgical interventions, the treated sites were frequently associated with residual BI and/or BOP scores.

### Efficacy of interventions

#### Alternative measures for biofilm removal

As an alternative to mechanical debridement, Er:YAG laser [42, 43], ultrasonic devices [44], and air-powder abrasive devices with glycine powder [45–47] were utilized to remove biofilm from contaminated implant surfaces. While the use of Er:YAG laser [42, 43] and an air-powered abrasive device with glycine powder [46, 47] led to significant improvements in BOP scores compared to mechanical debridement, the aforementioned alternative measures had no beneficial effect upon the changes in PD values. The use of an ultrasonic device failed to improve clinical treatment outcomes in terms of changes in BOP and PD when compared to mechanical debridement alone [44].

#### Adjunctive diode laser/aPDT

As an adjunct to mechanical therapy, the use of a diode laser resulted in comparable outcomes (i.e., BOP and PD changes) to the control group [49], whereas adjunctive aPDT therapy led to significantly higher PD and SBI reduction over a 6-month period compared to the control treatment (i.e., mechanical debridement) [48].

#### Adjunctive local antiseptics/antibiotics

In addition to mechanical debridement, application of local antibiotics (i.e., single [50, 52, 53] or repeated applications of minocycline microspheres [51]), CHX 1.0% gel (single [50] or repeated [51]), repeated application of CHX-containing chips [54, 55], or single subgingival placement of desiccant material [56] were investigated. Single application of minocycline microspheres in initial peri-implantitis cases (i.e., bone loss ≤ 3 mm) led to significantly higher PD reduction and comparable BOP changes [50], while repeated applications, on a contrary, yielded significantly greater BOP reduction, but similar PD changes [51] compared to the control sites (i.e., sites treated with mechanical debridement along with CHX 1.0% gel applications). Two RCTs reported similar changes in BOP values, but significantly higher PD improvements at implant sites treated with repeated CHX chips or single desiccant material application compared to placebo over 6 months [55, 56]. One study, however, failed to demonstrate any clinical beneficial effect in terms of BOP and PD changes of CHX chips over a 6-month period compared to the placebo group [54].

#### Adjunctive systemic antibiotics

Two RCTs investigated the potential benefits of the administration of systemic antibiotics along with mechanical debridement [57, 58]. Based on one RCT, prescribed systemic antibiotics (azithromycin 500 mg 3 day prior to treatment) along with mechanical debridement resulted in significant BOP and PD reduction (peri-implantitis definition: BO* p* + PD > 5 mm + bone loss > 2 mm) [57], whereas another RCT observed no beneficial effects of a combination of metronidazole 400 mg and amoxicillin 500 mg for BOP and PD changes in severe cases of peri-implantitis (i.e., BO* p* + PD > 5 mm + bone loss > 4 mm) [58].

#### Adjunctive probiotics

Contradictory findings were reported by 2 RCTs that evaluated the effects of the adjunctive use of probiotics for 6 months [59, 60]. In particular, one analysis failed to reveal any benefits of the adjunctive use of probiotic tablets and single local applications of probiotic drops upon the BOP and PD changes [60], whereas another RCT found similar BOP changes, but significant improvements in PD values following mechanical debridement along with systemic antibiotics in patients who also took probiotics for 6 months [59].

### Synthesis of results

#### Alternative measures for biofilm removal

**Fig. 3:**
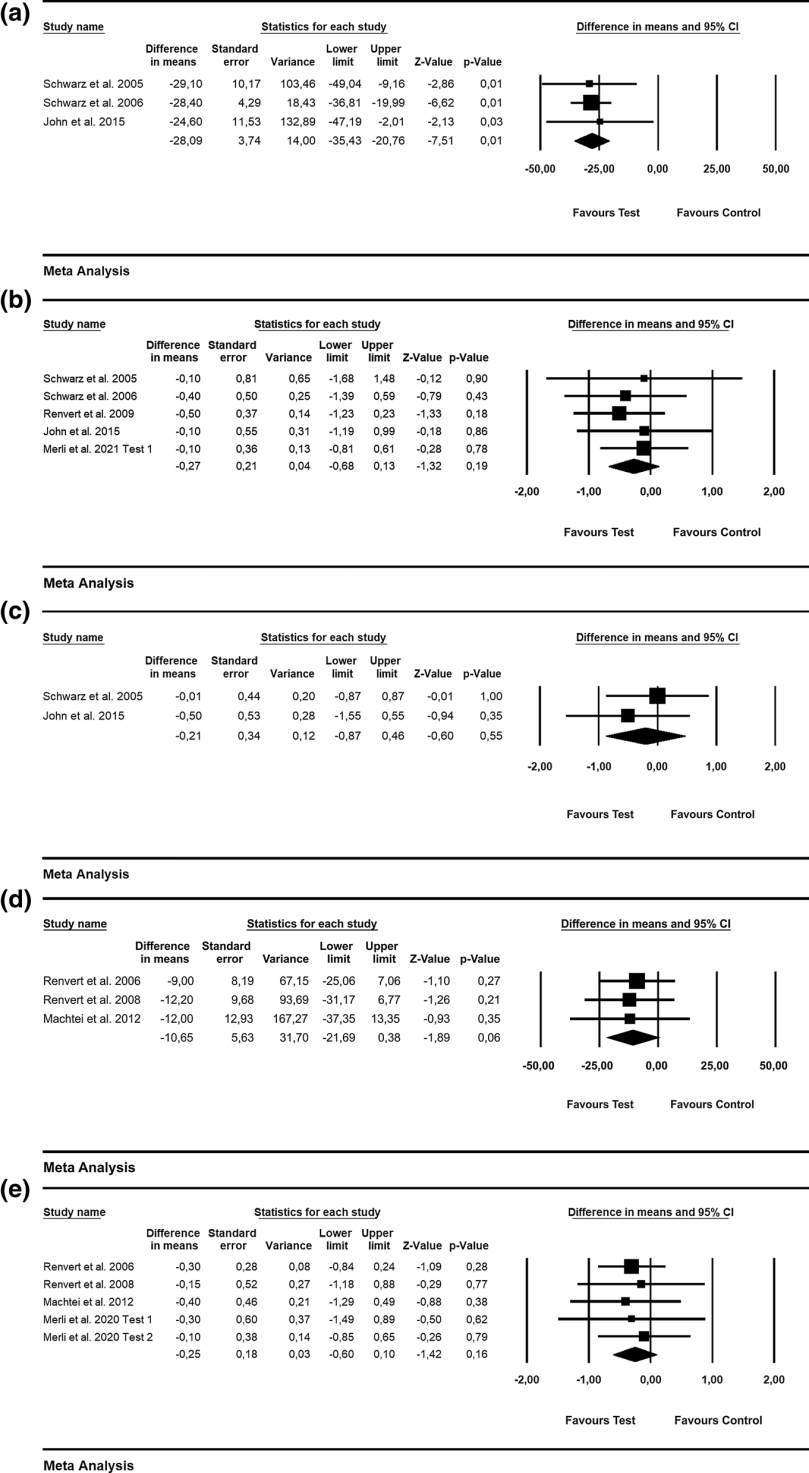

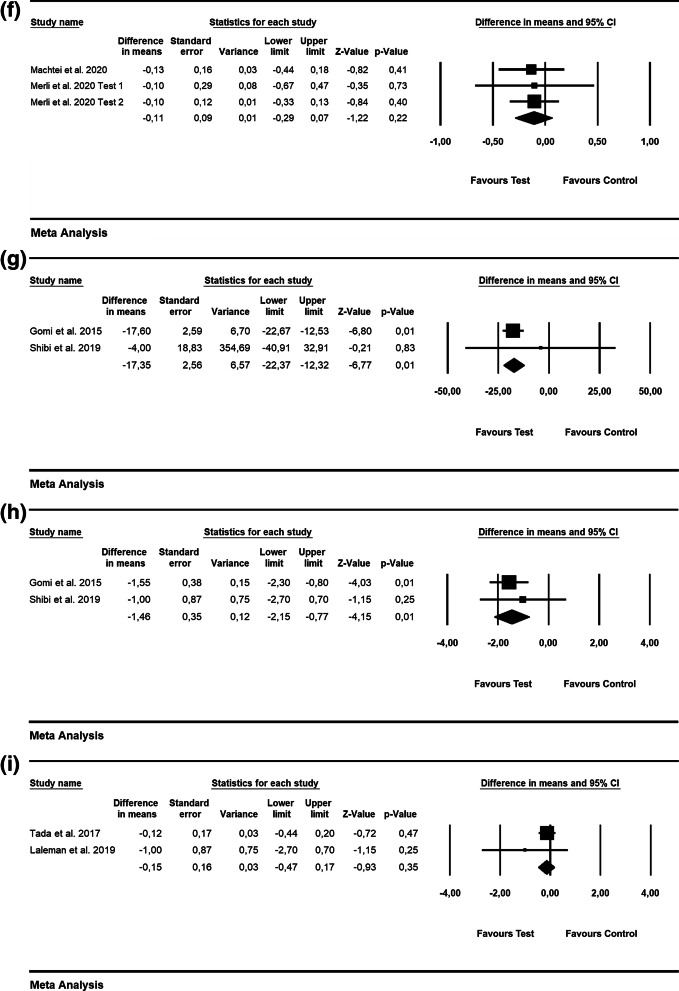
Forest plot indicating weighted mean difference (95% CI) in the reduction of assessed treatment outcomes following non-surgical treatment of peri-implantitis. **a** Alternative measures for biofilm removal (patient-level analysis)—BOP. **b** Alternative measures for biofilm removal (patient-level analysis)—PD. **c** Alternative measures for biofilm removal (patient-level analysis)—ML. **d** Adjunctive local antiseptic/antibiotic therapy (patient-level analysis)—BOP. **e** Adjunctive local antiseptic/antibiotic therapy (patient-level analysis)—PD. **f** Adjunctive local antiseptic therapy (patient-level analysis)—ML. **g** Adjunctive systemic antibiotics (patient-level analysis)—BOP. **h** Adjunctive systemic antibiotics (patient-level analysis)—PD. **i** Adjunctive probiotics (patient-level analysis)—PD

According to 3 RCTs, the WMD in BOP was − 28.09% [SE = 3.74; *p* = 0.01; 95% CI (− 35.43, − 20.76); unit of analysis: patient] in favor of alternative measures for biofilm removal (i.e., Er: YAG laser, air-powder abrasive device with glycine powder; p value for heterogeneity: 0.95, I^2^ = 0.0% = low heterogeneity) [42, 43, 47](Fig. 3a). The WMD in PD values was − 0.27 mm [SE = 0.21; *p* = 0.19; 95% CI (− 0.68, 0.13)]; unit of analysis: patient), thus not favoring the alternative measures used for biofilm removal (i.e., Er: YAG laser, air-powder abrasive device with glycine powder, ultrasonic device) as an adjunct to mechanical debridement (p value for heterogeneity: 0.938, I^2^ = 0.0% = low heterogeneity) (5 RCTs) [42–44, 47, 56](Fig. 3b).

Based on 2 RCTs, the WMD in ML was − 0.21 mm [SE = 0.34; *p* = 0.55; 95% CI (− 0.87, 0.46); unit of analysis: patient], suggesting no superiority of alternative measures for biofilm removal (p value for heterogeneity: 0.026, I^2^ = 80% = substantial heterogeneity) [42, 47] (Fig. 3c).

#### Adjunctive local antiseptics/antibiotics

Based on 3 studies, the estimated WMD in BOP values was − 10.65% [SE = 5.63; *p* = 0.06; 95% CI (− 21.69, 0.38)] (unit of analysis: patient), pointing to no beneficial effect of the local use of adjunctive antibiotics (i.e., minocycline microspheres) and local antiseptic (i.e., CHX) compared with mechanical debridement alone (p value for heterogeneity: 0.962, I^2^ = 0% = low heterogeneity; Fig. 3d) [50, 51, 54]. Based on 4 RCTs, the WMD in PD amounted to –0.25 mm [SE = 0.18; *p* = 0.16; 95% CI (− 0.60, 0.10)]; unit of analysis: patient), with adjunctive local antiseptic/antibiotic therapy not yielding higher PD reduction (p value for heterogeneity: 0.988, I^2^ = 0.0% = low heterogeneity; Fig. 3e) [50, 51, 54, 56]. The estimated WMD in ML was − 0.11 mm [SE = 0.09; *p* = 0.22; 95% CI (− 0.29, 0.07)]; unit of analysis: patient], thus indicating that the adjunctive local application of antiseptics did not lead to superior soft-tissue levels compared to mechanical debridement alone (p value for heterogeneity: 0.988, *I*^2^ = 0% = low heterogeneity; Fig. 3f) [55, 56].

#### Adjunctive systemic antibiotics

Based on 2 RCTs with 12 months of follow-up, the WMD in BOP and PD amounted to − 17.35% [SE = 2.56; *p* = 0.01; 95% CI (− 22.37, − 12.32)]; unit of analysis: patient) and − 1.46 mm [SE = 0.35; *p* = 0.01; 95% CI (− 2.15, − 0.77)]; unit of analysis: patient), thus supporting the favorable effect of adjunctive systemic antibiotics following mechanical debridement (p value for heterogeneity: 0.474, I^2^ = 0.0% and *p* = 0.562, I^2^ = 0.0% = low heterogeneity, Fig. 3g and h) [57, 58].

#### Adjunctive probiotics

The WMD in PD values was − 0.15 mm [SE = 0.16; *p* = 0.35; 95% CI (− 0.47, 0.17)]; unit of analysis: patient), not favoring adjunctive probiotics compared to mechanical debridement alone (2 RCTs) (*p* value for heterogeneity: 0.719, *I*^2^ = 0.0% = low heterogeneity, Fig. 3i) [59, 60].

### Surgical treatment of peri-implantitis

Ten RCTs (12 publications) reported on the non-reconstructive surgical treatment of peri-implantitis [18, 61–67, 71–74] and 9 studies (13 publications) reported on the surgical treatment of peri-implantitis employing adjunctive reconstructive measures (4 CCTs [69, 87–91], 5 RCTs [68, 82–86, 92]). The remaining 6 RCTs (7 publications) compared reconstructive peri-implantitis treatment over non-reconstructive approach [75–81, 93] and 2 RCTs (5 publications) reported on combined peri-implantitis therapy (i.e., implantoplasty + reconstructive therapy) (2 RCTs (5 publications) [19, 70, 94–96]) (Table 3).

**Table 3:** Included studies reporting on surgical peri-implantitis treatment

Publication	Design	Population	Case definition	Period	Test	Control	Mean (SD) outcome	Supportive therapy/comments
a)* Non-reconstructive surgery*								
Adjunctive and alternative measures for implant surface decontamination/systemic antibiotics								
Papadopoulos et al. (2015)	RCT, parallel	16 patients 12 females and 7 males. Mean age: 55 (8.7; range: 40–73) years 16 implants	BOP/ SUPP on probing + PD ≥ 6 mm and bone loss ≥ 2 mm	6 months	Mechanical debridement with plastic curettes + use of cotton swabs soaked in saline solution + use of a diode laser (low-power 980 nm)	Control Mechanical debridement with plastic curettes + use of cotton pellets soaked in saline solution	Subject level Test baseline: 81.2%; 6 months: 23.8% Control baseline: 93.8%; 6 months: 31.3% Significant reduction compared to the baseline (*p* < 0.05) No significant difference between groups (*p* > 0.05) PD Test baseline: 5.92 mm, 6 months: 4.44 mm Control baseline: 5.52 mm, 6 months: 4.31 Significant reduction compared to the baseline (*p* < 0.05) No significant difference between groups (*p* > 0.05)	Additional use of diode laser does not seem to have an extra beneficiary effect
Hallström et al. (2017)	RCT, parallel	31 patients Test: 15; mean age: 68.8 (25.0) years; female 75%; current smokers: 40%; tooth loss due to periodontitis: 47% Control: 16; mean age: 71 (7.7) years; female 63%; current smokers: 21%; tooth loss due to periodontitis: 53% 31 implants Test: 15 Control: 16	BOP/ SUPP on probing + PD ≥ 5 mm and bone loss ≥ 2 mm	12 months	OHI + mechanical debridement with curettes and cotton pellets soaked in saline + post-operative systemic antibiotics – Zithromax (Sandoz AS, Copenhagen, Denmark) 250 mg × 2 at the day of surgery, and 250 mg × 1 per day for 4 days	OHI + mechanical debridement with curettes and cotton pellets soaked in saline	Subject level BOP Test baseline: 100%; 12 months: 12.4 (9.2) % Control baseline: 100%; 12 months: 13.3 (11.1)% No significant difference between groups (*p* = 0.1) PD reduction Test: 1.7 (1.1) mm, * p* < 0.001 Control: 1.6 (1.5) mm, * p* < 0.001) No significant difference between groups (*p* = 0.5) RBL Test baseline: 4.6 (1.6) mm; 12 months: 4.0 (1.6) mm Control baseline: 4.9 (1.7), mm; 12 months: 4.5 (1.5) mm No significant difference between groups (*p* = 0.4)	During the study, participating individuals received professional prophylaxis every third month Adjunctive systemic azithromycin did not provide 1-year clinical benefits in comparison with access flap surgery alone
Albaker et al. (2018)	RCT, parallel	24 patients Tests: 11; mean age: 58.4 (8.0) years; 82% male; current smokers: 45% Control: 13; mean age: 61.5 (9.9) years; 69% male; current smokers: 54%	Bone loss ≥ 2 mm compared with previous examination or ≥ 3 mm (in the absence of previous radiograph) + PD ≥ 5 mm + BOP/SUPP	12 months	Access flap + implant cleaning with curettes and irrigation with sterile saline + aPDT (0.005% methylene blue photosensitizer, diode laser 670 nm 1 min + Augmentin 625 mg three times daily, 7 days + 0.2% CHX mouthrinse for 2 weeks	Access flap + implant cleaning with curettes and irrigation with sterile saline + Augmentin 625 mg three times daily, 7 days + 0.2% CHX mouthrinse for 2 weeks	Subject level BOP Test baseline: 35.9 (10.6)%, 12 months: 17.4 (5.5)% Control baseline: 26.5 (8.4)%, 12 months: 14.8 (3.1)% Between-group comparison: * p* = 0.22 PD Test baseline: 5.0 (1.2) mm, 12 months: 3.7 (1.1) mm, Control baseline: 5.4 (1.0) mm, 12 months: 3.9 (1.1) mm. Between group comparison: * p* = 0.51 Radiographic bone level Test baseline: 4.1 (1.4) mm, 12 months: 43.4 (1.4) mm; Control baseline: 4.5 (1.5) mm, 12 moths: 3.8 (1.4) mm Between-group comparison: * p* = 0.19	During the study, all patients received professional prophylaxis every third month Single application of aPDT does not provide additional benefit in improving clinical and radiographic parameters
Toma et al. (2019)	RCT, parallel	47 patients, 70 implants Test 1: 16 patients, 23 implants, mean age: 67.5 (12.9) years; 95% female; history of periodontitis: 73% patients Test 2: 16 patients, 23 implants; mean age: 61.7 (13.4) years; 81% female; history of periodontitis: 82% Control: 15 patients, 25 implants; mean age: 68.9 (15.8) years; 77% female; history of periodontitis: 84%	PD ≥ 5 mm + BOP/SUPP + radiographic bone loss ≥ 2 mm	6 months	Test 1: Access flap + mechanical debridement with plastic curettes + irrigation with sterile saline + air abrasive device with glycine powder + CHX mouthrinse 0.2% for 10 days Test 2: Access flap + mechanical debridement with plastic curettes + irrigation with sterile saline + titanium brush for 30 s with oscillating handpiece + CHX mouthrinse 0.2% for 10 days	Access flap + mechanical debridement with plastic curettes + irrigation with sterile saline + CHX mouthrinse 0.2% for 10 days	Implant level BOP Test 1 baseline: 59 (5.2)%; 6 months: 23 (2.3)%; p < 0.001 Test 2 baseline: 62 (4.7)%; 6 months: 16 (3.7)%; * p* < 0.001 Control: 54 (4.4)%; 6 months: 29 (3.4)%; * p* < 0.001 Significantly higher reduction in test 2 groups (*p* < 0.001) PD Test 1 baseline: 6.94 (1.29) mm; 6 months: 4.71 (1.24) mm; * p* < 0.001 Test 2 baseline: 6.45 (1.87) mm; 6 months: 3.98 (1.43) mm; * p* < 0.001 Control: 7.11 (1.15) mm; 6 months: 5.44 (0.69) mm; * p* > 0.001 Significantly great reduction in test 1 and test 2 groups (*p* < 0.001) RBL Test 1 baseline: 7.34 (1.29) mm; 6 months: 6.44 (1.46) mm; Test 2: 7.09 (1.23) mm, 6 months: 5.88 (1.3) mm Significantly less bone loss in test 2 group	3- and 6-months after surgery patients received professional supragingival cleaning Test treatments were more effective, but treatment success remained low
Cha et al. (2019)	RCT, parallel	46 patients Test: 24 patients/24 implants; mean age: 63 (range: 46–84) years; female 60% Control: 22 patients/ 22 implants; mean age: 60.2 (range: 40–83) years; female: 40%	Peri-implant bone loss > 2 mm + PD > 5 mm + BOP	6 months	OHI + mechanical debridement with titanium-coated curettes, metallic copper-alloy scaler tip, titanium brush and air abrasive device + adjunctive minocycline ointment Repeated applications after 1, 3 and 6 months	OHI + mechanical debridement with titanium-coated curettes, metallic copper-alloy scaler tip, titanium brush and air abrasive device + adjunctive placebo ointment Repeated applications after 1, 3 and 6 months	Subject level BOP/SUPP (%) change At the deepest site Test: 0.58 (0.50) Control: 0.32 (0.57); Intergroup comparison * p* = 0.102 Mean change: test: 0.49 (0.35), control: 0.31 (0.46); Between-group comparison: * p* = 0.141 PD changes At the deepest site Test: 3.58 (2.32) mm Control: 2.45 (2.13) mm; Between-group comparison: * p* = 0.094 Mean change Test: 2.68 (1.73) mm, control: 1.55 (1.86) mm, Between-group comparison: * p* = 0.039 RBL Test baseline: 6.33 (1.91) mm, 6 months: 7.05 (1.85) mm Control baseline: 5.16 (1.74) mm, 6 months: 5.47 (1.51) mm; * p* = 0.014 2.3-fold higher increase in test group (9.7 (0.56) mm vs control 0.31 (0.49) mm) Treatment success (PD < 5 mm + no BOP/SUPP + no further bone loss): Test: 55.7%, Control: 36.3%	All participants were recalled at 1, 3, and 6 mo to receive professional supragingival debridement and oral hygiene reinforcement Repeated local application of minocycline combined with access flap surgery provides significant benefits in terms of clinical parameters and radiographic bone fill, with a higher treatment success
De Waal et al. (2013)	RCT, parallel	30 patients Test: 15; mean age: 59.4 (14.0) years; female: 10; current smokers: 4; former smokers: 3; history of periodontitis: 6 Control: 15; mean age: 61.5 (10.0) years; female: 10; current smokers: 7; former smokers: 1; history of periodontitis: 5 79 implants machined, rough- and medium-rough surfaces Test: 15 patients, 31 implants Control: 15 patients, 48 implants	BOP/SUPP + PD ≥ 5 mm and bone loss ≥ 2 mm	12 months	OHI/mechanical debridement + resective therapy (apical re-positioned flap + bone re-contouring) + surface debridement using surgical gauzes soaked in saline + decontamination using 0.12% CHX + 0.05% cetylpyridinium chloride (CPC)	OHI/mechanical debridement + resective therapy (apical re-positioned flap + bone re-contouring) + surface debridement using surgical gauzes soaked in saline + decontamination using placebo solution	Implant level BOP (% of implants with BOP) Test baseline: 96.8 (30)%, 12 months: 96.8 (30)% Control baseline: 95.8 (46)%, 12 months: 94.7 (36)% No significant difference between groups (*p* = 0.965) PD Test baseline: 6.6 (1.6) mm, 12 months: 4.3 (2.1) mm Control baseline: 5.5 (1.4) mm, 12 months: 3.7 (0.8) mm. No significant difference between groups (*p* = 0.563) % of implants with SUPP Control baseline: 31.3 (15)%1; 12 months: 5.8 (6)% Test baseline: 64.5 (20)%; 12 months: 29.0 (9)% No significant difference between groups (*p* = 0.977) RBL Test baseline: 4.3 (2.1) mm, 12 months: 5.0 (2.5) Control baseline: 3.6 (1.9) mm, 12 months: 3.9 (2.0) No significant difference between groups (*p* = 0.949)	During follow‐up examinations, patients were re‐instructed in oral hygiene measures and implants and teeth were cleaned as necessary Implant surface decontamination with 0.12% CHX + 0.05% CPC in resective surgical treatment of peri-implantitis does not lead to superior clinical results
De Waal et al. (2015)	RCT, parallel	44 patients Test: 22; mean age: 58.6 (10.2) years; female: 17; current smokers: 7; former smokers: 1; history of periodontitis: 10 Control: 22; mean age: 60.5 (11.6) years; female: 14; current smokers: 6; former smokers: 5; history of periodontitis: 10 108 implants machined, rough- and medium-rough surfaces Test: 22 patients, 49 implants Control: 22 patients, 59 implants	BOP and/or SUPP on probing + PD ≥ 5 mm and bone loss ≥ 2	12 months	OHI/mechanical debridement + resective therapy (apical re-positioned flap + bone re-contouring) + surface debridement using surgical gauzes soaked in saline + decontamination using 0.12% CHX + 0.05% cetylpyridinium chloride	OHI/mechanical debridement + resective therapy (apical re-positioned flap + bone re-contouring) + surface debridement using surgical gauzes soaked in saline + decontamination using 2.0% CHX	Implant level BOP (% of implants with BOP) Test baseline: 98.0 (47)%, 12 months: 77.1 (37)% Control baseline: 94.9 (56)%, 12 months: 68.5 (37)% No significant difference between groups (*p* = 0.583) PD Test baseline: 4.7 (1.0) mm, 12 months: 3.0 (0.7) mm Control baseline: 5.0 (1.2) mm, 12 months: 2.9 (0.7) mm. No significant difference between groups % of implants with SUPP Baseline test:: 57.1 (28)%; 12 months: 10.4 (5)% Control baseline: 49.2 (29)%; 12 months: 1.9 (1)% No significant difference between groups (*p* = 0.222) RBL Test baseline: 4.0 (1.5) mm, 12 months: 4.3 (1.7) mm Control baseline: 4.1 (1.6) mm, 12 months: 4.1 (1.7) mm No significant difference between groups (*p* = 0.950)	During follow‐up examinations, patients were re‐instructed in oral hygiene measures and implants, and teeth were cleaned as necessary The use of a 2% CHX solution for implant surface decontamination during resective peri-implantitis therapy does not lead to improved clinical and radiographic results compared with a 0.12% CHX + 0.05% CPC solution
Carcuac et al. (2016, 2017)	RCT, parallel	67 patients Group 1: systemic antibiotics/implant surface decontamination with CHX: 27; mean age: 65.7 (range: 23–90) years; female: 20; smokers: 33.3%; history of periodontitis: 77.8% Group 2: systemic antibiotics/implant surface decontamination with saline: 25; mean age: 67.9 (range: 21–88) years; female: 17; smokers: 36%; history of periodontitis: 84% Group 3: no systemic antibiotics/implant surface decontamination with an CHX: 24; mean age: 64.6 (range: 27–81) years; female: 14; smokers: 33.3%; history of periodontitis: 87.5% Group 4: no systemic antibiotics/implant surface decontamination with saline: 24; mean age: 66.9 (range: 30–88) years; female: 14; smokers: 29.2%; history of periodontitis: 87.5% 121 implants: 25.6% non-modified, 74.4% modified surface Group 1 + 2: 68 implants Group 4 + 3: 53 implants	PD ≥ 6 mm + BOP/SUPP + bone loss > 3 mm	3 years	Debridement with titanium-coated curettes + Group 1 and group 3 decontamination with 0.2% CHX + Group 1 Amoxicillin 2*750 mg, 10 days, 3 days prior surgery	Debridement with titanium-coated curettes + Group 2 and 4 decontamination with saline for 2 min. + Group 2 Amoxicillin 2*750 mg, 10 days, 3 days prior surgery	Implant level BOP reduction 1 year Group 1: 39.1% Group 2: 34.8% Group 3: 44.4% Group 4: 51.4% No significant difference among groups (*p* < 0.05) 3 years: Presence of BOP/SUPP (%) Group 1: 66.2% Group 2: 52.8% Group 3: 70% Group 4: 32.3% PD reduction 1 year Group 1: 2.80 (1.87) mm Group 2: 3.44 (1.66) mm Group 3: 2.16 (1.79) mm Group 4: 1.69 (2.22) mm Significantly greater in group 2 than in groups 3 and 4 (*p* < 0.05) PD reduction 3 years Overall PD reduction compared to baseline: reduction of 2.73 ± 2.39 mm Group 1: 3.00 (2.44) mm Group 2: 2.38 (2.55) mm Group 3: 2.67 (2.48) mm Group 4: 2.90 (2.12) mm PD reduction was more pronounced at non-modified surface implants SUPP Baseline: mean: 68.7% Group 1: 72.3% Group 2: 65.2% Group 3: 67.3% Group 4: 70.3% After 1 year: Mean 17.4% Group 1: 13% Group 2: 6.5% Group 3: 22.2% Group 4: 31.4% RBL 3 years Group1: gain 0.32 ± 1.64 mm Group 2: loss − 0.51 ± 1.87 mm Group 3: loss − 0.28 ± 1.78 mm Group 4: gain 0.65 ± 0.86 mm	During the 12-mo follow-up period, supragingival polishing was performed and oral hygiene reinforced in 3-mo intervals The local use of chlorhexidine had no overall effect on treatment outcomes Potential benefits of systemic antibiotics are not sustained over 3 years
*Non-reconstructive therapy with implantoplasty*								
Romeo et al. (2005, 2007)	RCT, parallel	17 patients 22 implants rough surfaces Test: 10 patients, 19 implants Control: 7 patients, 16 implants	BOP/SUPP + PD > 4 mm horizontal peri-implant translucency	36 months	Full mouth disinfection/ mechanical debridement + resective therapy (apical re-positioned flap + bone re-contouring) + decontamination using metronida zole + tetracycline hydro chloride (3 min) + implantoplasty using diamond and Arkansas burs/silicone polishers + Amoxicillin 50 mg/kg/day for 8 days + CHX mouthrinse for 2 weeks	Full mouth disinfection/ mechanical debridement + resective therapy (apical re-positioned flap + bone re-contouring) + decontamination using metronidazole + tetracycline hydrochloride (3 min) + Amoxicillin 50 mg/kg/day for 8 days	Implant level mBI Test baseline: 2.83 (0.47), 3 years: 0.61 (0.67) Control baseline: 2.86 (0.35), 2 years: 2.33 (0.74) Between group comparison: Student’s t-value of + 9.61 PD Test baseline: 5.70 (1.69) mm, 2 years: 3.58 (1.06) mm, 3 years: 3.21 (0.56) mm Control baseline: 6.52 (1.62) mm, 2 years: 5.5 (1.47) mm. Significantly higher PD values in control group (Student’s t-value + 5.5) ML (recession) Test baseline: 0.5 (0.91) mm, 3 years: 1.96 (1.42) mm Control baseline: 0.23 (0.84) mm, 2 years: 1.64 (1.29) mm Between group comparison: Student’s t-value of + 9.61 Recession index in control group significantly lower (Student’s t-value of − 2.14) RBL mesial and distal Test baseline: 3.82 mm and 3.94 mm; 3 years: 3.81 mm and 3.94 mm Control baseline: 3.45 mm and 3.49 mm 3 years: 5.35 mm and 5.42 mm The mean variation of marginal bone level values mesial and distal Test: 0 and 0.001 mm (*p* > 0.05) Control: 1.44 and 1.54 mm (*p* < 0.05)	Implantoplasty was an effective treatment of peri-implant infection and peri-implantitis progression
Lasserre et al. (2020)	RCT, parallel	29 patients Smokers excluded 42 implants with modified surface Test: 15 patients; mean age: 62.3 (range: 42–74) years; female: 11; history of periodontitis: 13; 22 implants Control: 14 patients; mean age: 71 (range: 59–92) years; female: 11; history of periodontitis: 13; 20 implants	PD > 5 mm + bone loss ≥ 2 mm + BOP/SUPP	6 months	OHI + access flap + mechanical debridement with curettes + irrigation with sterile saline + implantoplasty + 0.1% CHX mouthrinse for 10 days	OHI + access flap + mechanical debridement with curettes + irrigation with sterile saline + air abrasive device with amino acid glycine powder + 0.1% CHX mouthrinse for 10 days p < 0.008	Implant level BOP Test baseline: 94.7 (10.7)%, 6 months: 33.3 (24.2)%, * p* < 0.008 Control baseline: 87.4 (22.3)%, 6 months: 26.3 (23.2)% PD Test baseline: 6.72 (1.78) mm, 6 months: 2.73 (1.59) Control baseline: 5.61 (1.56) mm, 6 months: 2.33 (1.49) mm ML (recession) Test baseline: 0.23 (0.48) mm, 6 months: 0.75 (0.71) mm, * p* < 0.008 Control baseline: 0.57 (0.85) mm, 6 months: 1.11 (0.89) mm RBL Test baseline: 4.73 (2.67) mm, 6 months: 4.47 (3.06), * p* < 0.008 Control baseline: 5.21 (2.06) mm, 6 months: 4.67 (2.05) mm, * p* < 0.008 No differences in any parameter between two groups (*p* > 0.008)	After 3 and 6 months careful professional supragingival cleansing was performed Implantoplasty is as effective as glycine air polishing
b) Studies comparing reconstructive therapy versus access flap surgery								
Wohlfahrt et al. (2012)	RCT, parallel	32 patients (13 female; 19 male) Test: 16; mean age: 65.0 (10.0) years; smokers: 6 (37.5%) Control: 16; mean age: 57.2 (12.3) years; smokers 10 (58.8%) 32 implants medium rough surfaces	PD ≥ 5 mm, BOP + intrabony defects ≥ 4 mm	12 months submerged healing for 6 months	Access flap surgery + mechanical debridement (titanium curettes) + conditioning using 24% ethylenediaminetetraacetic acid gel (2 min) + augmentation of intrabony defect components using porous titanium granules	Open flap surgery + mechanical debridement (titanium currettes) + conditioning using 24% ethylenediaminetetraacetic acid gel (2 min)	Implant level BOP (bleeding sites pro implant) reduction Test: 0.38 (2.1) % Control: 0.56 (2.9) % Not significant improvement compared to baseline No significant difference between groups (*p* = 0.60) PD reduction Test: 1.7 (1.7) mm Control: 2.0 (2.3) mm Significant improvement compared to baseline (*p* < 0.001) No significant difference between groups (*p* = 0.66) RDF Test: 57.0 (45.1) mm Control: − 14.8 (83.4) mm Significantly higher in test group (*p* < 0.001) Radiographic defect height reduction: Test: 2.0 (1.7) mm Control: 0.1 (1.9) mm Significantly higher in test group (*p* < 0.001)	Test group showed significantly better radiographic peri-implant defect fill compared with controls. Improvements in clinical parameters were seen in both groups, but no differences between groups were demonstrated
Andersen et al. (2017) (Wolhlfahrt et al. continuum)		12 patients 12 implants Test: 6 patients, 6 implants Control: 6 patients, 6 implants		7 years			Implant level PD changes Test baseline: 6.5 (1.9) mm, 7-years: 4.3 (2.4) mm Control baseline: 6.5 (2.3) mm, 7 years: 3.5 (1.2) mm RDF Mean radiographic osseous defect fill: test: 1.9 (2) mm control: 1.3 (1.4) mm Comparative statistical analysis was not performed	Follow-ups were handled by the referring dentists Surgical treatment of peri-implant osseous defects showed unpredictable results
Hamzacebi et al. (2015)	RCT, split-mouth design	19 patients, 38 implants Test: 19 implants Control: 19 implants Mean age: 60.98 (11.90) years	BOP/ SUPP + PD ≥ 5 mm + radiographic bone loss ≥ 2 mm	6 months	Access flap + mechanical debridement with PeriBrush + 4% pH 1 citric acid for 3 min or tetracycline chloride solution + postoperative mertonidazole (500 mg; 3 times per day; 7 days) + 0.12% CHX mouthrinse for 7 days	Access flap + mechanical debridement with PeriBrush + 4% pH 1 citric acid for 3 min or tetracycline chloride solution + intrabony defect fill with platelet-ruch fibrine (PRF) plugs and membranes + postoperative mertonidazole (500 mg; 3 times per day; 7 days) + 0.12% CHX mouthrinse for 7 days	Implant level BOP Test baseline: 79.31 (31.7)%; 6 months: 25.29 (14.51)%; * p* < 0.001 Control baseline: 65.47 (36.08)%; 6 months: 21.43 (16.57)%; * p* > 0.001 Between group comparison not conducted PD Test baseline: 6.13 (1.05) mm; 6 months: 3.30 (0.49) mm Control baseline: 5.78 (0.71) mm; 6 months:3.71 (0.42) mm Between-group comparison: * p* < 0.001 (higher reduction in test group) ML Test baseline: 0.62 (0.49) mm; 6 months: 0.14 (0.28) mm Control baseline: 0.83 (0.65) mm; 6 months: 1.04 (0.62) mm Between-group comparison: * p* < 0.001 (higher reduction in test grouo)	Supportive therapy NR PRF application led to better clinical results
Jepsen et al. (2016)	Multicenter RCT, parallel	63 patients (27 female, 36 male) 63 implants Test: 33 patients; mean age: 57.5 (12.6) years; current smokers: 11 (33.3%); former smokers: 9 (27.3%); history of periodontal treatment: 17 (51.5%); 33 implants Control: 30 patients; mean age: 59.1 (12.2) years; current smokers: 7 (23.3%); former smokers: 11 (36.7%); history of periodontal treatment: 20 (66.7%); 30 implants	PD ≥ 5 mm + BOP/SUPP + intraosseous circumferential three-wall defects ≥ 3 mm	12 months	Access flap + mechanical debridement with rotary titanium brush and H_2_O_2_ 3% (1 min) followed by rinsing with saline (60 s) + Titanium granules + Amoxicillin 500 mg 3 times/day + Metronidazole 400 mg 2 times/day, 8 days, starting 1 day before surgery	Access flap + mechanical debridement with rotary titanium brush and H_2_O_2_ 3% (1 min) followed by rinsing with saline (60 s) + Amoxicillin 500 mg 3 times/day + Metronidazole 400 mg 2 times/day, 8 days, starting 1 day before surgery	Subject level BOP reduction Test: 56.1 (30.5)% Control: 44.9 (38.2)% Significant reduction compared to baseline (*p* < 0.001) No significant difference between groups (*p* > 0.05) PD reduction Test: 2.8 (1.3) mm Control: 2.6 (1.4) mm Significant reduction compared to baseline (*p* < 0.001) No significant difference between groups (*p* > 0.05) SUPP reduction Test: 23.2 (32.8) % Control: 25.6 (32.7) % Significant reduction compared to baseline (*p* < 0.001) No significant difference between groups (*p* > 0.05) Radiographic defect height reduction: mesial/distal: test: 3.61 (1.96)/3.56 (2.07) mm control: 1.05 (1.42)/1.04 (1.34) mm Significantly higher in test group (*p* < 0.0001) RDF mesial/distal: test: 79.00 (29.85)%/74.22 (36.33)% control: 23.11 (46.28)%/21.89 (30.16)% Significantly higher in test group (*p* < 0.0001)	Patients were recalled at 6 wk and 3, 6, 9, and 12 mo after surgery for professional oral hygiene procedures with supragingival debridement and hygiene instructions provided as needed Test group showed significantly enhanced radiographic defect fill compared with control group. Similar improvements according to clinical measures were obtained after both surgical treatment modalities
Renvert et al. (2018)	RCT, parallel	41 patients 41 implant Test: 21 patients; female: 13; 21 implants Control: 20 patients; female: 9; 20 implants	PD ≥ 5 mm + BOP/SUPP + marginal bone loss, defined as a crater like defect ≥ 3 mm	12 months	Access flap + mechanical debridement with titanium curettes + decontamination with 3% H_2_O_2_ + application of bovine-derived deproteinized bone particles + Zitromax (Sandoz AS; Copenhagen, Denmark) 500 mg day one and 250 mg days 2–4	Access flap + mechanical debridement with titanium curettes + decontamination with 3% H_2_O_2_ + Zitromax (Sandoz AS; Copenhagen, Denmark) 500 mg day one and 250 mg days 2–4	Implant level BOP Test baseline: 100%, 12 months: 47.6% Control baseline: 100%, 12 months: 35% No difference between groups (*p* = 0.41) PD Test baseline: 6.5 (1.9) mm, 12 months: 2.9 (1.4) mm, * p* > 0.001 Control baseline: 6.7 (1.8) mm, 12 months: 4.2 (2.8) mm, * p* > 0.001 Significantly greater reduction in the test group (*p* < 0.01) ML (mid-buccal recession) Test: 1.2 m Control: 1.9 mm No difference between groups (*p* = 0.76) RDF Test: 0.7 (0.9) mm Significant compared to the baseline (* p* = 0.004) Control: 0.2 (0.2) mm Not significant compared to baseline (*p* = 0.10) Successful treatment outcome (defect fill ≥ 1.0 mm, PPD values at implant ≤ 5 mm, no BOP, and no SUPP): Test: 9/21 (42.9%) patients Control: 1/20 (5.0%) patients; Between-group comparison: * p* < 0.01	Based on individual needs, professional prophylaxis was performed every 3rd month Successful treatment outcome using a bone substitute was more predictable when a composite therapeutic endpoint was considered
Isehed et al. (2016, 2018)	RCT, parallel	23 patients 23 implants Smokers inlcuded Test: 10 patients, 10 implants Control: 13 patients, 13 implants	PD ≥ 5 mm + BOP/ SUPP + angular bone loss ≥ 3 mm	5 years	Access flap + mechanical debridement with ultrasonic device and titanium hand instruments + cotton pellets soaked in sodium chloride + application of Emdogain (EMD) (0.3 ml) + non-submerged healing	Access flap + mechanical debridement with ultrasonic device and titanium hand instruments + cotton pellets soaked in sodium chloride + non-submerged healing	Implant level BOP-positive sites 5 years Test: 5/11 (55.6%) implants Control: 2/9 (40%) implants Presence of SUPP: Test baseline: 9/15 (60%); 1 year: 1/15 (7%) Control baseline: 6/14 (43%); 1 year: 1/14 (7%) SUPP at 3 years Test: 2/13 (20%) implants Control: 3/12 (33%) implants MBL changes 5 years Test: 4.1 mm Control: 3.3 mm Change: test: + 1.4 mm Control: + 1.3 mm *p* = 0.90	Between 1 and 5 years after the peri‐implant surgical treatment, supportive therapy was performed based on individual needs at the specialist clinic or by the patient's general dental clinic, usually with 3‐ or 6‐month intervals Test group showed increased bone levels 12 months after treatment
Renvert et al. (2021)	RCT	66 patients 66 implants Test: 34; female 54%; age: 62.2 (10.2) years Smokers 8 (22%) Control: 32; female 50%; age: 62.9 (10) years; smokers: 9 (26%) Heavy smokers (> 10 cig./day) excluded	PD ≥ 5 mm + BOP/SUPP + radiographic bone loss ≥ 3 mm + intraosseous defect component of at least 3 mm depth and a circumference at least 270 ◦ detected intraoperatively	1 year	Access flap + debridement with titanium curettes + titanium brush + decontamiantion with 3% H_2_O_2_ 1 min + saline rinsing + defect fill with bovine bone mixed with bloos + bilaminar collagen membrane + postoprative antibiotics (Azithromycin 500 mg, 4 days; + CHX 0.2% moutrinse for 3 weeks	Access flap + debridement with titanium curettes + titanium brush + decontamiantion with 3% H_2_O_2_ 1 min + saline rinsing + postoprative antibiotics (Azithromycin 500 mg, 4 days; + CHX 0.2% moutrinse for 3 weeks	Subject level BOP (severity of blleding pro implant) Test baseline: 1.3 (0.9), 12 months: 0.4 (0.6) Control baseline: 1.4 (1.0); 12 months: 0.5 (0.6); Between-group comparison: * p* = 0.992 PD Test baseline: 6.7 (1.5) mm, 12 months: 4.8 (1.5); Control baseline: 6.8 (1.3); 12 months: 4.5 (1.5) mm Between-group comparison: * p* = 0.578 SUPP (sites per implant) Test baseline: 1.8 (1.4); 12 months: 0.3 (0.9); Control baseline: 1.6 (1.5), 12 months: 0.3 (0.9) Between-group comparison: * p* = 0.879 ML (recession) Test baseline: 0.4 (1.8) mm, 12 months: 0.8 (1.2) mm Control basleine: 0.6 (0.9); 12 months: 1.4 (1.5) mm Between-group comparison: * p* = 0.136 RDF at the deepest site Test: 2.7 (1.3) mm Control: 1.4 (1.2) mm; Between-group comparison: * p* < 0.001 Mean RDF Test: 2.3 (1.2)mm Control: 1.1 (1.1) Between-group comparison: * p* = 0.001	Oral hygiene insructions were provided after 3, 6, 9 and 12 months following the surgery Reconstructive therapy resulted in significantly more RDF. No difference in clinical paramenetrs was noted
*c) Reconstructive therapy*								
Adjunctive and alternative measures for implant surface decontamination following reconstructive therapy								
Deppe et al. (2007)	CCT, parallel	16 patients 32 implants machined, rough- and medium-rough surfaces Test: 9 patients, 17 implants Control: 7 patients, 15 implants	PD ≥ 5 mm, BOP + progressive vertical bone loss	5 years	3 weeks prior to surgery: CHX gel applications (0.3%) Group 2 OHI + access flap surgery + air abrasive device + carbon dioxide laser (cw mode, 2.5 W, 12 × 5 s) decontamination + beta tricalcium phosphate + cortical bone chips harvested from the retromoar area (50:50) + nonresorbable synthetic barrier membrane	3 weeks prior to surgery: CHX gel applications (0.3%) Group 4 OHI + access flapb surgery + air abrasive device + beta tricalcium phosphate + cortical bone chips harvested from the retromoar area (50:50) + nonresorbable synthetic barrier membrane	Implant level SBI Test baseline: 0.5 (0.8), 5 years: 2.1 (1.4) Control baseline: 1.2 (0.6), 5 years: 1.9 (1.0) PD Test baseline: 5.0 (1.3) mm, 5 years: 2.5 (1.4) mm Control baseline: 4.8 (1.4) mm, 5 years: control: 2.5 (1.1). No significant difference between the groups (*p* > 0.05) Radiographic DIB (distance from the implant shoulder to the first bone contact) Test baseline: 2.3 (0.9) mm 5 years: 4.5 (1.2) mm Control baseline: 4.1 (0.9) mm, 5 years: 4.7 (1.1) mm No significant difference between the groups (*p* > 0.05)	Over the 5-year period, if plaque and bleeding scores indicated poor oral hygiene, remotivatino and reinstruction of OHI were performed There seems to be no difference between laser and conventional decontamination
Isler et al. (2018a)	RCT, parallel	41 patients 60 implants Test: 20 patients; mean age: 54.4 (8.08) years; female: 9; current smokers: 5 (25%); history of periodontitis: 9 (45%); 30 implants Control: 21 patients; mean age: 54.18 (10.36) years; female: 10; current smokers: 6 (28.5%); history of periodontitis: 8 (38%); 30 implants	≥ 2 mm marginal bone loss + BOP/SUP with or without deepening of PDs	12 months	Access flap + mechanical debridement with titanium curettes + irrigation with saline (3 min.) + ozone application + bovine bone mineral mixed with pieces of concentrated growth factors (CGF) + coverage with CGF membranes + Amoxicillin (500 mg) + Metronidazole (500 mg) 3 times/day for 1 week	Access flap + mechanical debridement with titanium curettes + irrigation with saline (3 min.) + bovine bone mineral mixed with CGF + coverage with CGF membranes + Amoxicillin (500 mg) + Metronidazole (500 mg) 3 times/day for 1 week	Implant level BOP Test baseline: 96.6 (10.5), 12 months: 15.8 (19.1), * p* < 0.001 Control baseline: 97.5 (10.06), 12 months: 25 (21.7), * p* < 0.001 No difference between groups (*p* = 0.575) PD Test baseline: 6.27 (1.42) mm, 12 months: 2.75 (0.7) mm, * p* < 0.001 Control baseline: 5.73 (1.11) mm, 12 months: 3.34 (0.85) mm, * p* < 0.001 No difference between the groups (*p* = 0.158) ML (recession): Tests baseline: 0.12 (0.14) mm, 12 months: 0.48 (0.75) mm, * p* < 0.01 Control baseline: 0.25 (0.42) mm, 12 months: 0.55 (0.64) mm, * p* < 0.01 No difference between groups (*p* = 0.753) RDF Test: 2.32 (1.28) mm Control: 1.17 m (0.77) mm Significantly higher fill in test group (*p* = 0.02)	The patients were re-evaluated at 1, 3, 6, 9, and 12 months postoperatively and supportive care was given at the same time points Higher radiographic defect fill in the test group
Reconstruction of the defect with different bone fillers, with and without a membrane								
Khoury et al. (2001)	CCT, parallel	25 patients; mean age: 48.2 (6.3) years; 22 female 41 implants Test 1: 20 implants Test 2: 9 implants Control: 12 implants	Bone loss > 50% of implant length + intrabony crater-form defect	3 years	Test 1 Access flap + decontamination with 0.2% CHX, citric acid (pH = 1) (1 min.) and rinsed with H_2_O_2_ + Test 1 autogenous bone + non-resorbable membrane Test 2 autogenous bone + resorbable membrane + submerged healing + Antibiotics administered 4 weeks prior to surgery (for 1 week), and later starting 1 day and finishing 7 days after surgery according to the individual susceptibility test results	Access flap + decontamination with 0.2% CHX, citric acid (pH = 1) (1 min.) and rinsed with H_2_O_2_ + autogenous bone + submerged healing + Antibiotics administered 4 weeks prior to surgery (for 1 week), and later starting 1 day and finishing 7 days after surgery according to the individual susceptibility test results	Implant level PD changes Test 1: 5.4 (3.0) mm Test 2: 2.6 (1.6) mm Control: 5.1 (2.7) mm Significant improvement compared to baseline in all groups (*p* > 0.001) Significantly less improvement in test 2 group compared to test 1 and the control (*p* ≤ 0.05) Radiographic vertical intrabony defect height reduction: Test 1: 2.8 (3.1) mm Test 2: 1.9 (3.2) mm Control: 2.4 (2.7) mm Significantly less improvement in test 2 group compared to baseline (*p* = 0.102) No difference among the groups (*p* ≤ 0.05)	The patients wereenrolled in a supportive maintenance program and monitored on a 3- to 6-month recall schedule including repeated oral hygiene instructions and a full-mouthtooth cleaning according to their individual needs 17 out of 29 barrier-treated implants (58.6%) were compromised by early post-therapy complication (e.g., dehiscence, exposure, fistula, or sequester formation)
Schwarz et al. (2006, 2008, 2009)	RCT, parallel	20 patients; 14 female; mean age: 54.4 (12.5) years; 1 patient light smoker (< 10 cig./day) 21 implants Test: 9 patients, 9 implants Control: 10 patients, 11 implants	PD > 6 mm + BOP/SUPP + intrabony component > 3 mm	4 years	OHI + initial non-surgical therapy Access flap surgery + mechanical debridement (plastic curettes) + nanocrystalline hydroxyapatite paste + non-submerged healing	OHI + initial non-surgical therapy Access flap surgery + mechanical debridement (plastic curettes) + bovine-derived xenograft + native collagen barrier membrane + non-submerged healing	Subject level BOP reduction Test: 32% Control: 51% PD reduction Test: 1.1 (0.3) mm Control: 2.5 (0.9) mm BOP and PD reductions significantly higher at control sites	A supragingival professional implant/tooth cleaning and reinforcement of oral hygiene were performed at 1, 3, 6, 12, 18, 24, 30, 36, 42, and 48 months after treatment Long-term outcome obtained in test group without barrier membrane must be considered as poor
Aghazadeh et al. (2012)	RCT, parallel	45 patients 71 implants medium-rough surfaces Test: 23 patients; mean age: 67.0 (7.5) years; smokers: 69.6%; 37 implants Control: 22 patients; mean age: 70.1 (6.2) years; smokers: 40.9%; 34 implants	PD ≥ 5 mm + BOP/SUPP + radiographic bone loss ≥ 2 mm + angular peri-implant bone defect ≥ 3 mm	12 months	Access flap surgery + mechanical debridement (titanium instruments) + decontamination using hydrogen peroxide 3% cortical bone chips harvested from the mandibular ramus + resorbable synthetic barrier Membrane + Azithromycin 2 × 250 mg 1 day, 1 × 250 mg 2–4 days	Access flap surgery + mechanical debridement (titanium instruments) + decontamination using hydrogen peroxide 3% bovine-derived xenograft + resorbable synthetic barrier membrane + Azithromycin 2 × 250 mg 1 day, 1 × 250 mg 2–4 days	Implant level BOP reduction Test: 50.4 (5.3)% Control: 44.8 (6.3)% No significant difference between the groups (*p* > 0.05) PD reduction Test: 3.1 (0.2) mm Control: 2.0 (0.2) mm Significantly higher in the test group (*p* < 0.01) SUPP reduction Test: 25.2 (4.3)% Control: 11.5 (5.2)% Significantly higher in the test group (*p* < 0.01) RDF Test: 1.1 (0.3) mm Control: 0.2 (0.3) mm Significantly higher in test group (*p* < 0.05)	Six weeks after surgery the first supportive therapy was given, and the subjects were enrolled in a maintenance program with visits everythird month. Allexisting teeth and implants werecleaned using a rubber cup and alow-abrasive paste Bovine xenograft provided more radiographic bone fill than authogenous bone
Roos-Jansaker et al. (2007, 2011, 2014)	CCT, parallel	25 patients 45 implants Test: 13 patients; mean age: 64.9 (7.5) years; current smokers: 10 (76.9%); former smokers: 2 (15.4%); 23 implants Control: 12 patients; mean age: 65.7 (7.4) years; current smokers: 8 (66.7%); former smokers: 3 (25%); 22 implants	Bone loss > 3 threads (≥ 1.8 mm) one-to-four intrabony defect + BOP and/or SUPP	5 years	Removal of the suprastructure Access flap surgery + debridement + decontamination using 3% H_2_O_2_ + algae-derived xenograft + resorbable synthetic barrier membrane + non-submerged healing + systemic antibiotic medication (Amoxicillin + Metronidazole for 10 days)	Removal of the suprastructure Access flap surgery + debridement + decontamination using 3% H_2_O_2_ + algae-derived xenograft + non-submerged healing + systemic antibiotic medication (Amoxicillin + Metronidazole for 10 days)	Implant level PD reduction at the deepest site Test: 3.0 (2.4) mm Control: 3.3 (2.0) mm No significant difference between the groups (*p* = 0.60) ML(recession changes at the deepest site) Test: − 1.6 (1.5) mm Control: − 1.7 (2.1) mm No significant difference between the groups (*p* = 0.89) RDF Test: 1.5 (1.2) mm Control: 1.1 (1.2) mm No significant difference between the groups (*p* = 0.24)	The participants were then enrolled in a maintenance program with visits every third month. At these visits, full‐mouth plaque scores were obtained. Re‐instruction in oral hygiene procedures was performed as necessary. Teeth and implants were cleaned using a rubber cup and a low‐abrasive paste Additional use of a membrane did not improve the outcome
Güler et al. (2017)	CCT, parallel	24 patients (9 female, 15 male). mean age: 45.36 (14.1) years 35 implants Test: 18 patients; 19 implants Control: 6 patients, 16 implants Light smokers included (< 10 cig.7 day): Test: 3 (18.75%) Control: 3 (50%)	PD > 5 mm + BOP/SUPP Class Ib° defects (vestibular dehiscence + circumferential bone resorption) Class Ic°° defects (vestibular dehiscence + circumferential bone resorption) Class Id defects (circumferential bone resorption)	6 months	OHI + access flap + mechanical cleaning with rotating titanium brush + titanium granules + PRF (platelet-rich fibrin membrane) + non-submerged healing + systemic antibiotics Amoxicillin clavulanate 2 × 1000 mg/day, 7 days	OHI + access flap + mechanical cleaning with rotating titanium brush + xenograft + resorbable collagen membrane + PRF (platelet-rich fibrin membrane) + non-submerged healing + systemic antibiotics Amoxicillin clavulanate 2 × 1000 mg/day, 7 days	Implant level BOP Test baseline: 50.17 (25.19)%, 6 months: 24.32 (11.22)% Control baseline: 63.51 (24.38)%, 6 months: 33.00 (15.51)% Significantly higher reduction in test group (*p* = 0.02) PD Test baseline: 5.28 (1.06) mm, 6 months: 3.34 (0.82) mm Control baseline: 4.72 (1.02) mm, 6 months: 3.34 (0.82) mm No significant difference between groups (*p* = 0.698) ML (recession) Test baseline: 0.01 (0.003) mm, 6 months: 0.42 (0.58) mm Control baseline: 0.208 (0.452) mm, 6 months: 0.51 (0.48) mm No significant difference between groups (*p* = 0.476) RDF Test: 1.74 (0.65) mm Control: 1.05 (0.54) mm Significantly higher reduction in test group (*p* = 0.006)	Radiographic bone filling was significantly higher in the test group
Isler et al. (2018b)	RCT, parallel	52 patients 105 implants Test: 26 patients; female: 10; current smokers: 6; history of periodontitis: 11; 52 implants (23% on-modified, 77% modified) Control: 26 patients; female: 15; current smokers: 9; history of periodontitis: 13; 52 implants (19.2% non-modified, 80.8% modified)	Bone loss ≥ 2 mmbased on baseline radiograph + BOP /SUPP	12 months	OHI + supra/subgingival mechanical debridement 4–6 weeks prior to surgery Access flap + mechanical debridement with titanium curettes and saline-soaked cotton gauses + bovine bone filler + concentrated growth factor (CGF) membrane + systemic antibiotics Amoxicillin 500 mg + metronidazole 500 mg, 3 times a day, 1 week + 0.12% CHX mouthrinse 2 weeks	OHI + supra/subgingival mechanical debridement 4–6 weeks priot to surgery Access flap + mechanical debridement with titanium curettes and saline-soaked cotton gauses + bovine bone filler + collagen membrane + systemic antibiotics Amoxicillin 500 mg + metronidazole 500 mg, 3 times a day, 1 week + 0.12% CHX mouthrinse 2 weeks	Implant level BOP Test baseline: 97.12 (10.79)%, 12 months: 35.58 (30,.14)%, * p* < 0.001 Control baseline: 97.12 (8.15)%, 12 months: 29.81 (30.02), * p* < 0.001 Between-group comparison: * p* = 0.503 PD Test baseline: 5.92 (1.26) mm, 12 months: 3.71 (1.09) mm, * p* < 0.001 Control baseline: 5.41 (1.16) mm, 12 months: 2.70 (0.80) mm, * p* < 0.001 Between-group comparison: * p* = 0.001 ML (recession) Test baseline: 0.04 (0.20) mm, 12 months: 0.25 (0.39) mm, * p* = 0.007 Control baseline: 0.06 (0.20) mm, 12 months: 0.27 (0.44) mm, * p* = 0.026 Between-group comparison: * p* = 0.925 RDF Test: 1.63 (1.0) mm Control: 1.98 (0.75) mm, * p* = 0.154 Treatment success (PD < 5 mm + no BOP/SUPP, no further bone loss): Test: 26.9% implants Control: 42.3% implants	All patients were enrolled in postoperative maintenance care programs at three different time points during the study periods (3, 6, and 9 months). Supragingival/mucosal mechanical debridement and reinforcement of oral hygiene were performed during postoperative period. When necessary localized subgingival/mucosal instrumentation was done except for the area of surgery Control group showed better results
Polymeri et al. (2020)	RCT, parallel	24 patients, 24 implants Test: 13; mean age: 57.3 (15.1) years; female: 5 (38%); smokers: 2 (15%); history of periodontal treatment: 6 (46%) Control: 11; mean age: 65.5 (11.2) years; female: 6 (55%); smokers: 3 (27%); history of periodontal treatment: 4 (36%)	Bone loss ≥ 3 mm + PD ≥ 5 mm + BOP/SUPP + intra-osseous defect component ≥ 3 mm at the deepest part and presence of at least three walls	12 months	Access flap + mechanical debridement with titanium curettes + decontamination with 3% H_2_O_2_ 1 min + xenogrfat (EndoBone) + non-submerged healing + systemic antibiotics Amoxicillin 500 mg twice a day, 8 days, starting 1 day prior to surgery + 4 weeks mouthrinse with 0.12% CHX	Access flap + mechanical debridement with titanium curettes + decontamination with 3% H_2_O_2_ 1 min + xenogrfat (BioOss) + non-submerged healing + systemic antibiotics Amoxicillin 500 mg twice a day, 8 days, starting 1 day prior to surgery + 4 weeks mouthrinse with 0.12% CHX	Subjest level BOP Test basleine: 100 (0.0)%, 12 months: 50 (10.2)%, * p* < 0.001 Control baseloine: 100 (0.0)%, 12 months: 45.5 (33.2)%, * p* < 0.001 Between-group comparison: * p* = 0.670 PD Test basleine: 7.1 (1.2)%, 12 months: 3.4 (0.5)%, * p* < 0.001 Control baseloine: 7.0 (1.8)%, 12 months: 3.4 (0.6)%, * p* < 0.001 Between-group comparison: * p* = 0.910 Radiographic defect depth Test baseline:; 5.9 (1.8) mm, 12 months: 2.9 (1.3), * p* < 0.001 Control baseline: 4.9 (0.9) mm, 12 months: 2.4 (0.6) mm, * p* < 0.001 Bestween-group comparison: * p* = 0.183 RBL Test baseline: 4.9 (1.1) mm, 12 months: 2.1 (1.3)mm, * p* < 0.001 Control baseline: 5.3 (1.2) mm, 12 months: 3.1 (1.3) mm, * p* < 0.001 Between-group comparison: * p* = 0.073 Treatment success (PD ≤ 5 mm + no BOP/SUP + no further bone loss): test: 13%, control: 18% of patients	Patients were recalled at 6 weeks and 3, 6, 9, and 12 months after the surgery for professional oral hygiene procedures that included supragingival debridement and polishing with a rubber cup and a low-abrasive paste Test and control groups showed comparable outcomes
*d) Combined therapy*								
Schwarz et al. (2011, 2012, 2013, 2017)	RCT, parallel	15 patients 11 females, 4 males; median age: 63 years Heavy smokers (≥ 10 cigarettes/day) excluded 15 patients Test: 6 patients, 6 implants Control: 9 patients, 9 implants	PD ≥ 6 + BOP/SUPP + intrabony component > 3 mm + supracrestal component > 1 mm	7 years	Initial non-surgical therapy + OHI Access flap + Er:YAG laser device (cone-shape glass fiber tip) at 11.4 J/cm2 + implantoplasty at buccally and supracrestally exposed implant parts + bovine-derived xenograft + native collagen membrane + non-submerged healing	Initial non-surgical therapy + OHI Access flap mechanical debridement with plastic curettes and saline-soaked cotton gauses + implantoplasty at buccally and supracrestally exposed implant parts + bovine-derived xenograft + native collagen membrane + non-submerged healing	Subjest level BOP reduction Test: 86.66 (18.26)% Control: 89.99 (11.65)% Significant improvement compared to baseline (*p* < 0.001) PD reduction Test: 0.74 (1.89) mm Control: 2.55 (1.67) mm Significant improvement compared to the baseline (*p* < 0.001) ML (reduction of recession) Test: 1.36 (1.04) mm Control: 0.49 (0.92) mm	A supragingival professional implant/tooth cleaning and reinforcement of oral hygiene wereperformed at 1, 3, and 6 months after therapy. Afterwards, recall appointments to provide a professionally administered plaque removal and reinforcement of oral hygiene were scheduled on an annual basis Combined surgical therapy of advanced peri-implantitis was not influenced by the initial method of surface decontamination
De Tapia et al. (2019)	RCT, parallel	30 patients Heavy smokers (≥ 10 cigarettes/day) excluded 30 patients Test: 15 patients; mean age: 65.53 (10.29) years; female: 11 (73.3%); light smokers: 6 (40%); 15 implants Control: 15 patients; mean age: 55.47 (11.75) years; female: 9 (60%); light smokers: 4 (26.7%); 15 implants	PD ≥ 6 mm + BOP/SUPP + bone loss > 30% of the implant surface + intrasurgically osseous defect with at least two bone walls and depth of 3 mm of intrabony component	12 months	Initial non-surgical therapy: OHI Access flap + implantoplasty supracrestally with diamond burs and Arkansas stone + debridement using plastic ultrasonic scalers + rinsing with H_2_O_2_ 3% + titanium brush with an oscillating low speed + non-submerged healing + combination of 500 mg Amoxicillin and 500 mg Metronidazole 3 times a day, for 7 days	Initial non-surgical therapy: OHI Access flap + implantoplasty supracrestally with diamond burs and Arkansas stone + debridement using plastic ultrasonic scalers + rinsing with H_2_O_2_ 3% + non-submerged healing + combination of 500 mg Amoxicillin and500 mg Metronidazole 3 times a day, for 7 days	Subject level BOP Test baseline: 100%, 12 months: 79% Control baseline: 100%, 12 months: 55%, between-group comparison: * p* = 0.147 PD Test baseline: 6.16 (1.27) mm; 12 months: 3.31 (0.72) Control: 6.17 (0.98) mm; 12 months: 3.87 (0.81) mm Between-group comparison: * p* = 0.04 SUPP Test baseline: 43%; 12 months: 0% Control baseline: 47%; 12 months: 23%; * p* = 0.053 ML (recession) Test: 0.4 (0.45) mm Control: 0.6 (0.62) mm; Between-group comparison: * p* = 0.374 RBL Test: 2.51 (1.21) mm, Control: 0.73 (1.26) mm; Between-group comparison: * p* = 0.003 RDF: Test: 81 (22)% Control: 52 (55)%; Between-group comparison: * p* = 0.111	Patients were seen at weekly intervals for the first 4 weeks to monitor healing and, then, at 3‐month intervals during the first year The additional use of a titanium brush during combined treatment of peri-implantitis resulted in statistically significant benefits in terms of PD reduction

*RCT* randomized clinical trial, *OHI* oral hygiene instructions, *BOP* bleeding on probing, *PD* probing depth, *SUPP* suppuration, *BI* bleeding index, *mBI* modified bleeding index, *RBL* radiographic bone level, *RDF* radiographic bone defect fill, *ML* soft-tissue level, *H*_*2*_*O*_*2*_ hydrogene peroxide

Follow-up periods among the included studies varied from 6 months (6 studies), 1 year (12 studies), 3 to 4 years (4 studies), to 5 and 7 years (5 studies). Peri-implantitis was commonly defined by the presence of BOP/SUPP and a presence of radiographic bone loss, with the threshold values of ≥ 2 mm or > 3 mm being most frequently used. In fact, the majority of the studies (13 studies) reporting on reconstructive and combined peri-implantitis therapy indicated the presence of intrabony peri-implant defect configuration (Table 3). Twenty studies (29 publications) reported on patient engagement into a regular supportive therapy following the surgery [18, 19, 61, 63–68, 71, 74, 76–78, 80–87, 89–92, 94–96].

### Efficacy of interventions

#### Adjunctive and alternative measures for implant surface decontamination following non-reconstructive treatment

Over the 6-month follow-up period, alternative measures for implant surface decontamination, including a titanium brush and an air-powder abrasive with glycine powder, were more effective in reducing signs of inflammation, as shown by a higher reduction in BOP and PD values over the implant sites treated with the conventional decontamination method (i.e., plastic curettes) [67]. Furthermore, sites treated with a titanium brush revealed significant improvements in marginal bone levels compared to the implants treated with either an air powder abrasive device or plastic curettes (i.e., control group). Nonetheless, as addressed by the authors, treatment success (i.e., PD ≤ 5 mm, no BOP, no bone loss ≥ 5 mm) was rarely obtained irrespective of the decontamination protocol (i.e., plastic curettes: 22% of implants; air-powder abrasive: 33% of implants; titanium brush: 33% of implants) [67].

Based on 2 RCTs, the adjunctive use of either a PDT or diode laser failed to reveal any beneficial clinical effect with respect to BOP and PD changes throughout the 6-month period [62, 63]. Three RCTs investigated the additional use of 0.2% CHX solution for implant surface decontamination [18, 61], and adjunctive decontamination using 0.12% CHX + 0.05% cetylpiridinium chloride (CPC) versus placebo [65], or 0.12% CHX + 0.05% CPC versus 2.0% CHX [66]. Over 1- to 3-year follow-up periods, the adjunctive use of the aforementioned antimicrobials as a part of implant surface decontamination protocol did not lead to improved clinical (i.e., BOP and PD) or radiographic outcomes compared with the respective controls [18, 61, 65, 66].

#### Adjunctive implantoplasty following non-reconstructive treatment

Two RCTs (3 publications) assessed the clinical efficacy of implantoplasty used as an adjunct to non-reconstructive therapy [71–73]. In particular, data from a 6-month RCT pointed to no differences in clinical (i.e., BOP and PD changes) and radiographic parameters between implant sites treated with either implantoplasty or air polishing with glycine powder [71]. A 3-year RCT, contrarily, indicated that adjunctive implantoplasty enhanced implant survival rates, significantly reduced PDs, SUPP, and BI, and was associated with stable marginal bone levels compared to the control sites, where bone loss amounted to 1.45–1.54 mm [72]. However, sites treated with implantoplasty resulted in significantly more soft tissue recession (test group: 2.3 [1.45] mm, control group: 1.64 [1.29] mm) [72, 73] (Table 3).

#### Adjunctive local and systemic antibiotics following non-reconstructive treatment

Based on 1 RCT, the repeated local applications of antibiotics (i.e., minocycline oinment 1, 3 and 6 months postoperatively) lead to significant benefits in terms of greater mean PD reduction and radiographic marginal bone levels compared to the control implant sites (i.e., mechanical debridement and air-powder polishing), while changes in BOP/SUPP were comparable between test and control groups [64].

Two RCTs investigated the potential beneficial effect of systemic antibiotics following non-reconstructive peri-implantitis treatment [18, 61, 74]. Specifically, over a 1-year period, the adjunctive administration of postoperative systemic antibiotics lead to similar clinical (i.e., changes in BOP and PD), radiographic (i.e., RBL) or microbiological treatment outcomes compared to the control group [74]. Based on the results of another RCT, a positive effect of systemic antibiotics on the success of treatment (i.e., PD ≤ 5 mm, no BOP/SUPP, bone loss ≤ 0.5 mm) during a 1-year period was observed only for implants with modified surface characteristics [61]. The benefits of the systemic antibiotic regimen, however, did not last through the 3-year follow-up, leading to similar changes in BOP, SUPP, PD and RBL values [18].

#### Adjunctive and alternative measures for implant surface decontamination following reconstructive therapy

Adjunctive use of ozone therapy for implants as part of implant surface decontamination protocol along with reconstructive peri-implantitis treatment over a 1-year period resulted in significantly greater peri-implant bone defect fill compared to decontamination with sterile saline solution (2.32 mm vs. 1.17 mm, respectively), whereas clinical outcomes (i.e., changes in BOP and PD) were comparable between test and control groups [68]. After 5 years of follow-up period, adjunctive application of CO_2_ laser provided similar clinical (i.e., changes in BOP and PD) and radiographic treatment outcomes to the conventional decontamination approach (i.e., air polishing) [69].

#### Adjunctive and alternative measures for implant surface decontamination following combined therapy

Use of a titanium brush as an adjunct treatment to surface decontamination protocol (i.e., debridement with ultrasonic scaler + rinsing with H_2_O_2_ 3%) after 1 year resulted in significantly greater PD reduction compared to control implant sites, while BOP changes were similar in both treatment groups [70]. After 7 years of follow-up, implant surface decontamination by means of Er:YAG monotherapy following combined peri-implantitis therapy led to similar BOP and PD changes as to implant sites where conventional decontamination protocols were used (i.e., mechanical debridement + saline-soaked cotton gauze) [19] (Table 3).

#### Reconstruction of peri-implant bone defects with different bone fillers

After 12 months of healing, significantly higher RDF and mean PD reduction were obtained at peri-implantitis defects filled with xenogenic bone filler particles in comparison with autogenous bone, whereas BOP changes were similar for both reconstruction approaches [82]. In comparison with synthetic bone filler (i.e., nanocrystalline hydroxyapatite particles), the use of a bovine-derived xenograft after 4 years led to significantly greater BOP and PD improvements [83]. Increased RDF and higher BOP reduction were detected at implants treated with porous titanium granules compared with xenograft, whereas PD reduction and clinical attachment changes did not differ between the treatment groups [88]. The comparison of the 2 xenograft materials over a 12-month period led to similar treatment outcomes as depicted by similar changes in the BOP, PD, and RDF values, as well as the treatment success (defined as PD ≤ 5 mm + no BOP/SUPP + no further bone loss) [86].

#### Reconstruction of peri-implant bone defects with and without a membrane

One 3-year CCT reported significantly lower PD reduction and less RDF at implant sites treated with autogenous bone along with non-resorbable membrane compared with those treated with either autogenous bone alone or in combination with resorbable membrane [87]. Peri-implantitis defects reconstructed using bovine bone along with a collagen membrane after 4 years showed significantly lower BOP and PD values compared with the implant sites treated with synthetic bone filler (i.e., nanocrystalline hydroxyapatite particles) [83]. Another 5-year CCT indicated no beneficial effect of the adjunctive use of a synthetic resorbable membrane along with xenogenic bone substitute particles, as the changes in PD, ML, and RDF were comparable between the treatment groups [90]. Furthermore, the comparison of the 2 membranes (i.e., concentrated growth factor membrane and collagen membrane) applied over the xenogenic bone filler after 1 year resulted in similar BOP changes and comparable RDF, whereas a greater PD reduction was registered at sites treated with the adjunctive collagen membrane [85].

#### Reconstructive therapy versus non-reconstructive surgery

Six RCTs (7 studies) assessed the clinical efficacy of reconstructive therapy over access flap surgery [75–81, 93] (Table 4 b). One to 7 years following the treatment, a significantly higher RDF was observed at the implant sites treated with either titanium granules or xenograft bone filler, as compared with the control sites (i.e., access flap surgery) [75, 76, 78, 80, 93]. On a contrary, as noted in 2 RCTs with 6-month and 5-year follow-up periods, the adjunctive use of either enamel matrix protein (EMD) or platelet-rich fibrin (PRF) had no beneficial effect upon RDF changes [77, 79, 81]. In terms clinical outcomes, after 1- to 7-years of follow-up, the PD and BOP changes did not differ between the implant sites treated with either titanium porous granules or xenogenic bone filler particles and those obtained at the control sites [75, 76, 80, 93]. Two studies, in contrast, reported greater PD reduction after 1 year at implants treated with either adjunctive xenogenic bone substitute or PRF, while changes on BOP values were similar between the test and control groups [78, 79]. Regarding changes to soft-tissue levels, the use of xenogenic bone filler particles did not lead to superior ML outcomes after 1 year [78, 80], whereas implant sites treated with adjunctive PRF after 6 months showed significantly lower ML values as compared to the controls (test: 0.14 mm, control: 1.04 mm) [79].

### Synthesis of results

#### Adjunctive implantoplasty following non-reconstructive treatment

**Fig. 4:**
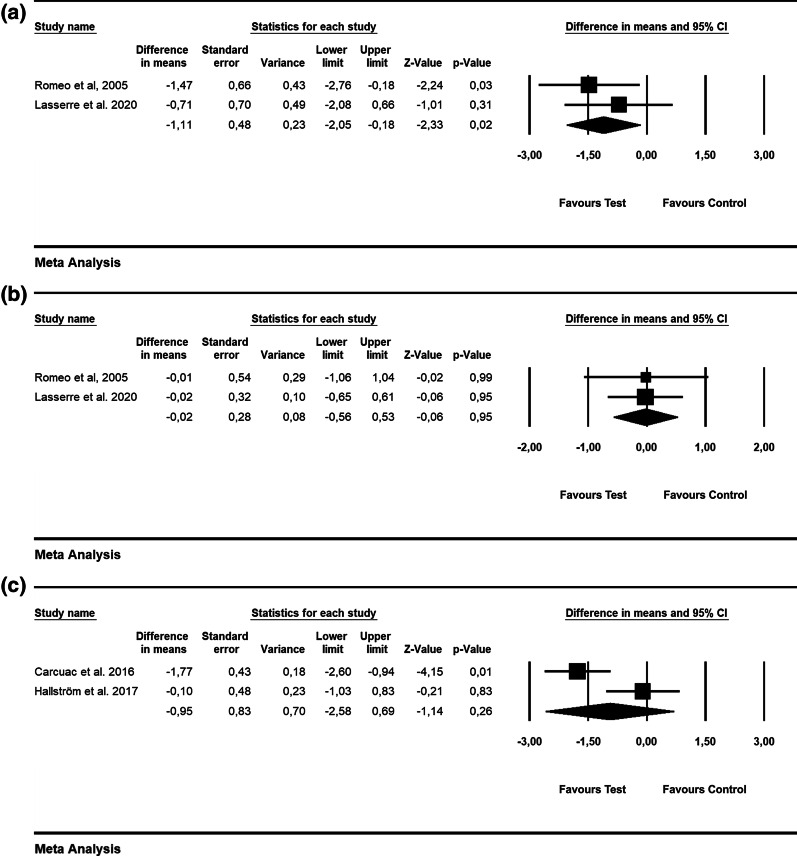
Forest plot indicating weighted mean difference (95% CI) in the changes of clinical outcomes following non-reconstructive surgical treatment of peri-implantitis. **a** Adjunctive implantoplasty (implant-level analysis)—PD. **b** Adjunctive implantoplasty (implant-level analysis)—ML. c Adjunctive systemic antibiotics (implant-level analysis)—PD

A meta-analysis based on 2 RCTs indicated the WMD in PD of − 1.11 [SE = 0.48; *p* = 0.02; 95% CI (− 2.05, − 0.18)] (unit of analysis: implant); p value for heterogeneity: 0.429, I^2^ = 0% = low heterogeneity), thus suggesting higher PD reduction at implant sites treated with implantoplasty [71–73]. The WMD in ML amounted to − 0.02 [SE = 0.28; *p* = 0.95; 95% CI (− 0.56, 0.53); unit of analysis: implant], pointing to no significant difference between test and control groups in terms of soft-tissue level changes (p value for heterogeneity: 0.99, I^2^ = 0% = low heterogeneity) [71, 72] (Fig. 4a and b).

#### Adjunctive systemic antibiotics following non-reconstructive treatment

Based on 2 RCTs with 1 year of follow-up, WMD in PD amounted to − 0.95 [SE = 0.83; *p* = 0.26; 95% CI (− 2.58, 0.69)]; unit of analysis: implant), thus not favoring administration of adjunctive systemic antibiotics following non-reconstructive peri-implantitis treatment (p value for heterogeneity: 0.009, I^2^ = 85.39% = substantial heterogeneity; Fig. 4c).

#### Reconstructive therapy versus non-reconstructive surgery

**Fig. 5:**
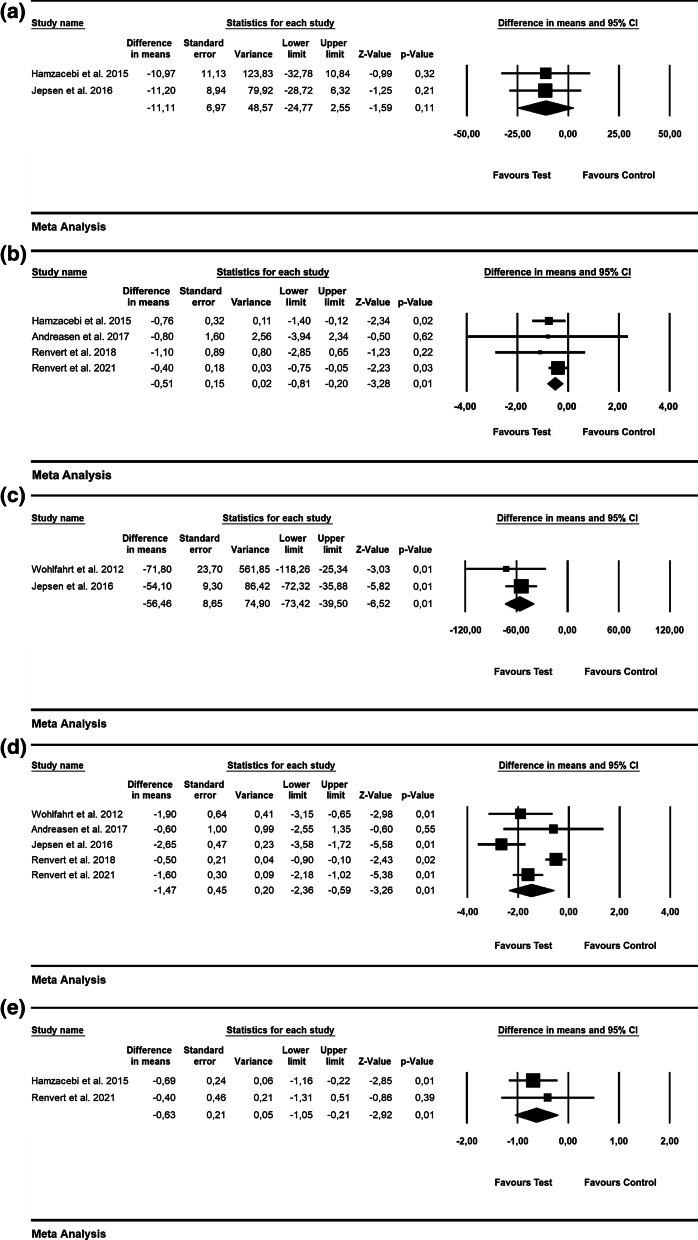
Forest plots depicting weighted mean differences (95% CI) in the changes of primary and secondary outcomes between reconstructive and non-reconstructive peri-implantitis surgical treatment. **a** BOP reduction (implant-level analysis). **b** PD (mm; implant-level analysis). **c** RDF (%; implant-level analysis). **d** Radiographic defect reduction (mm; implant-level analysis). **e** ML (implant-level analysis)

The WMD in BOP reduction was − 11.11% [SE = 5.97; *p* = 0.11; 95% CI (− 24.77, 2.55)] (unit of analysis: implant), indicating no differences between reconstructive and non-reconstructive treatment approaches (p value for heterogeneity: 0.983, I^2^ = 0% = low heterogeneity) [79, 97] (Fig. 5a). The WMD in PD revealed a significant difference between the test and control groups (WMD = − 0.51 mm [SE = 0.15; *p* = 0.01; 95% CI (− 0.81, − 0.20)] (unit of analysis: implant) that favored adjunctive reconstructive approaches (p value for heterogeneity: 0.28, I^2^ = 21% = low heterogeneity) [78–80, 93] (Fig. 5b). The WMD in RDF amounted to − 56.46% [SE = 8.65; *p* = 0.01; 95% CI (− 73.42, − 39.50)] (unit of analysis: implant), pointing to a higher defect fill in the test group (p value for heterogeneity: 0.487, I^2^ = 0% = low heterogeneity) [75, 76] (Fig. 5c). Based on data from 4 RCTs, the WMD in reduction of radiographic defects was − 1.47 mm [SE = 0.45; *p* = 0.01; 95% CI (− 2.36, − 0.59)] (unit of analysis: implant), suggesting significantly higher reduction in the test group (p value for heterogeneity: 0.389, I^2^ = 0% = low heterogeneity) (Fig. 5d). The WMD in ML was − 0.63 mm [SE = 0.21; *p* = 0.01; 95% CI (− 1.05, − 0.21)] (unit of analysis: implant), favoring reconstructive measures (p value for heterogeneity: 0.579, I^2^ = 0 = low heterogeneity) [79, 80] (Fig. 5e).

### Risk of bias in individual studies

Of the included 55 RCTs, 23 appeared to have an overall unclear risk of bias, 18 were judged to have a high risk of bias, and the remaining 14 had a low risk of bias (Additional file 2a).

Four of the included CCTs had an overall serious risk of bias, and the remaining 3 had an overall critical risk of bias (Additional file 2b).

## Discussion

The present systematic review aimed to evaluate the efficacy of alternative and adjunctive measures compared to conventional treatment of peri-implant mucositis and peri-implantitis. In total, 55 RCTs and 7 CCTs were included in the analysis. Of those, 18 reported on non-surgical treatments of peri-implant mucositis, and 17 and 27 reported on non-surgical and surgical peri-implantitis treatments, respectively.

The primary goal of peri-implant mucositis treatment has been established as the resolution of inflammation as evidenced by the absence of BOP [98]. Based on the current data synthesis, the investigated alternative measures for biofilm removal (i.e., glycine powder air polishing and chitosan brushes) and adjunctive measures (i.e., diode laser, aPDT, local antiseptic therapy, probiotics, home care mouth rinse) failed to improve BOP scores over mechanical debridement alone. In terms of PD values, while the adjunctive use of local antiseptics (i.e., CHX and sodium hypochlorite) along with mechanical debridement led to significantly greater PD reduction (WMD = − 0.23 mm, *p* = 0.03, respectively), similar PD improvements were noted regardless of the implementation of the aforementioned adjunctive measures for biofilm removal, aPDT, probiotics or home care mouthrinse. The present findings partially align with the results of previous systematic reviews and meta-analyses according to which adjunctive measures for treating peri-implant mucositis (i.e., antiseptics, local and systemic antibiotics, air-abrasive devices) failed to improve the efficacy of professionally administered plaque removal in reducing clinical signs of inflammation, as shown by comparable changes in BOP and PD values [13, 99]. However, the calculations in those analyses were based on pooled data from clinical studies that employed both local and systemic adjunctive measures (i.e., local and systemic antibiotics), which in turn might at least partially explain the aforementioned discrepancies [13, 99]. Taken together, the use of investigated adjunctive and alternative measures were not found to be superior in resolving peri-implant mucositis**,** thus supporting recent consensus statements suggesting that non-surgical mechanical instrumentation in conjunction with oral hygiene reinforcement is a standard-of-care intervention for the management of peri-implant mucositis [4, 12, 100].

According to recent recommendations, results of peri-implantitis treatment should be assessed following a healing period of at least 6 months and should be based on a composite outcome, including parameters such as bone fill, peri‐implant soft tissue recession, PD, BOP, and SUPP [97, 98]. The present analysis included clinical studies reporting on peri-implantitis treatment outcomes with an observation period of at least 6 months [97]. In contrast to peri-implant mucositis, non-surgical treatment of peri-implantitis including alternative measures for biofilm removal (i.e., glycine powder air polishing, Er:YAG laser) yielded higher BOP reduction compared to conventional measures (i.e., mechanical debridement with or without CHX; WMD = − 28.09%; *p* = 0.01), whereas these improvements were not observed in PD scores (WMD = − 0.27 mm; *p* = 0.19). Additionally, BOP and PD reductions were not improved by the adjunctive use of local antiseptics/antibiotics (BOP WMD = − 10.65%, *p* = 0.06; PD WMD = − 0.25 mm, *p* = 0.16), nor by the use of probiotics (PD WMD = − 0.15, *p* = 0.35). Furthermore, soft-tissue level changes following treatment were similar regardless of whether alternative biofilm removal measures (WMD = − 0.21, *p* = 0.55) or local antiseptics (WMD = − 0.11, *p* = 0.22) were employed. The aforementioned findings corroborate the results of one former meta-analysis, which reported significantly greater BOP reduction at implant sites treated with either adjunctive local antibiotic therapy (i.e., minocycline microspheres) or alternative plaque removal measures (i.e., Er:YAG laser or glycine powder air polishing) over respective control treatments [13]. Further analysis revealed a significantly higher reduction in BOP and PD values throughout the 12-month period with administration of systemic antibiotics along with the mechanical debridement (WMD = − 17.35%; *p* = 0.01 and WMD =  − 1.46 mm; *p* = 0.01, respectively). However, this estimation is based on only 2 RCTs, one of which included only severe cases of peri-implantitis (case definition: BOP + PD > 5 mm + bone loss > 4 mm) and found no beneficial effect of systemic antibiotics (amoxicillin + metronidazole) following non-surgical peri-implantitis treatment [58]. Likewise, one recent RCT reported no clinical and microbiological benefits of systemic antibiotics (amoxicillin + metronidazole) along with non-surgical treatment of peri-implantitis (case definition: bone loss ≥ 2 mm + BOP/SUPP + PD ≥ 5 mm) compared to mechanical debridement and local CHX irrigation after 3 months, thus concluding that the administration of systemic antibiotics should not be routinely recommended [101]. Notably, the majority of the included studies reported on residual BOP/BI scores following non-surgical peri-implantitis treatment, and disease resolution (i.e., absence of BOP and further bone loss) was obtained in 14% to 47% of the cases 6 to 12 months after the treatment [45, 52, 56]. Therefore, in line with earlier findings, non-surgical treatment of peri-implantitis seems to have limited efficacy in predictably resolving inflammation, thus supporting the necessity of surgical treatment in the majority of patients diagnosed with peri-implantitis [12, 102]. Nonetheless, according to the recent recommendations, non-surgical therapy should always precede surgical intervention in treating peri-implantitis [102].

Due to heterogeneity in reporting, no quantitative analysis was feasible for the impact of implantoplasty on the resolution of peri-implant tissue inflammation (i.e., BOP/SUPP changes) following surgical non-reconstructive peri-implantitis treatment. Nonetheless, based on the present findings, though implant sites treated with or without implantoplasty resulted in similar postoperative changes in soft-tissue levels (WMD = − 0.02 mm, *p* = 0.95), significantly higher PD reduction was found at sites treated with adjunctive implantoplasty (WMD = − 1.11 mm, *p* = 0.02). With respect to the rationale for administration of systemic antibiotics following non-reconstructive peri-implantitis treatment, no differences in PD improvements were found between the test and control groups throughout the 12-month period (WMD = − 0.95 mm, *p* = 0.26). This latter finding supports the results of a 3-year RCT, which after 1 year observed positive effects of systemic antibiotics on the non-reconstructive peri-implantitis treatment success (i.e., PD ≤ 5 mm, no BOP/SUPP, bone loss ≤ 0.5 mm) at implants with a modified surface [61]. However, those benefits were not sustained over a 3-year period, thus not supporting the benefits of the systemic antibiotic regimen [18].

Six RCTs evaluated the potential beneficial effect of reconstructive peri-implantitis treatment over control approaches (i.e., access flap). In particular, meta-analyses identified a significantly higehr RDF (WMD = − 56.46%, *p* = 0.01), radiographic defect resolution (WMD = − 1.47 mm; *p* = 0.01) and greater PD reduction at the implant sites treated with adjunctive reconstructive measures compared to the controls (− 0.51 mm, *p* = 0.01). However, in terms of resolution of mucosal inflammation (i.e., BOP changes), no differences could be detected between the test and control groups (WMD = − 11.11%; *p* = 0.11). Those findings slightly contradict the results of previous meta-analyses that reported on radiographic bone-level gains and RDF for reconstructive treatment approaches over access flap surgery, whereas similar values were reported for PD and BOP changes [103, 104]. Nonetheless, noteworthy are the discrepancies among the studies included in the present meta-analysis with respect to grafting materials with different radiopacities and osteoconduction properties, which might have influenced the obtained outcomes. Upon further data analysis, implant sites treated with adjunctive reconstructive measures yielded lower postoperative changes soft-tissue recession compared to sites treated via access flap surgery (WMD = − 0.63 mm; *p* = 0.01). This latter outcome corroborates the results of one recent meta-analysis, according to which use of adjunctive reconstructive measures lead to significantly lower increase in mucosal recession when compared to non-reconstructive peri-implantitis treatment (WMD = − 1.35 mm, *p* = 0.038) [104].

Along these lines, it is worthwhile to note that the treatment outcomes of peri-implant mucositis and peri-implantitis might be influenced by the surface characteristics of the abutment and/or implant. In fact, clinical data have reported greater BOP reduction following the treatment of experimentally induced peri-implant mucositis lesions at implants with machined abutments, as compared to the modified surfaced abutments [105]. As documented by the previous analyses, significantly better outcomes were obtained after surgical non-reconstructive therapy of peri-implantitis at implants with non-modified surfaces compared to modified surfaces, as shown by the superior BOP, PD reductions and superior bone-level preservation at non-modified surfaced implants [15, 18]. Additionally, more favorable clinical and radiographic outcomes of surgical reconstructive peri-implantitis therapy were documented for moderately rough surfaced implants compared to rough surfaced implants [106]. The results of a majority of the studies included in the present analysis were based on implants with modified surfaces. Thus, due to the limited data availability, subanalyses to validate the extent to which implant/abutment surface properties might have influenced the treatment outcomes of peri-implant mucositis and peri-implantitis were not feasible.

Several limitations of the present systematic review must be addressed. First, a majority of the included studies lacked true control groups and therefore could not be included in the quantitative analysis. Second, most studies included in meta-analysis had follow-up periods that were limited to 12 months, thus the present findings are valid only for the short-term outcomes. Further, the present analysis pooled clinical studies that applied different case definitions for peri-implant mucositis and peri-implantitis. In fact, depending on the individual protocols used, factors such as peri-implant bone defect morphology and severity of the disease have previously been found to be influencing factors for the outcomes following surgical treatment of peri-implantitis [105–108]. Finally, peri-implant soft-tissue conditions (i.e., presence or lack of keratinized mucosa), patients` adherence to supportive therapy following peri-implant mucositis and peri-implantitis treatment as well as patient-related factors, such as smoking habits, systemic conditions (i.e., diabetes) and intake of different medications may also be important factors contributing to the outcomes of therapy. However, in the present analysis, due to inconsistencies in reporting among the studies, potential effects of these factors on treatment outcomes of peri-implant diseases could not be investigated.

## Conclusions

Alternative and adjunctive measures provided no beneficial effect in resolving peri-implant mucositis, while alternative measures were superior in reducing BOP values following non-surgical peri-implantitis treatment. Adjunctive reconstructive measures along with surgical peri-implantitis treatment were beneficial regarding radiographic bone-defect fill/reduction, PD reduction and lower soft-tissue recession, although they did not improve the resolution of mucosal inflammation. Systemic antibiotics added no benefits to surgical non-reconstructive peri-implantitis treatment outcomes. The potential benefits of resective measures upon inflammation resolution need to be further investigated.

## Supplementary Information

**Additional file 1:** Excluded studies.

**Additional file 2:** a. Risk‐of‐bias summary of included randomized trial according to the RoB2 risk of bias tool for randomized studies. b. Risk‐of‐bias summary according to the ROBINS-I risk of bias tool for non-randomized studies.

## Data Availability

Not applicable.

## Abbreviations

RCT: Randomized clinical trial
OHI: Oral hygiene instructions
BOP: Bleeding on probing
mBOP: Modified bleeding on probing index
PD: Probing depth
SUPP: Suppuration
BI: Bleeding index
mBI: Modified bleeding index
aPDT: Antibacterial photodynamic therapy
