# Supplementary material for: Efficacy of alternative or adjunctive measures to conventional non-surgical and surgical treatment of peri-implant mucositis and peri-implantitis: a systematic review and meta-analysis
Source: Int J Implant Dent. 2021 Nov 15;7:112. doi: 10.1186/s40729-021-00388-x (PMC8593130; doi:10.1186/s40729-021-00388-x)
Supplement: Supplementary file 1 — Additional file 1: Excluded studies. [file 40729_2021_388_MOESM1_ESM.docx]

| **Study** | **Reason for exclusion** |
| --- | --- |
| Studies on peri-implant mucositis |  |
| Schenk et al. 1997 | < 5 patients per treatment group |
| Alzoman et al. 2020  Hallström et al. 2015 | Results presented only graphically |
| Kashefimehr et al. 2017  Tenore et al. 2020 | Pooled cases of peri-implant mucositis and peri-implantitis |
| Strooker et al. 1998  Alqahtani et al. 2016 | Case definition of peri-implant mucositis not reported  Case definition of peri-implant disease not reported |
| De Siena et al. 2014 | Home care plaque control protocol |
| Flichy-Fernandez et al. 2015  De Tapia et al. 2019 | Compared outher factors than different treatment protocols |
| José González-Serrano et al. 2021 | Follow-up period < 3 months |
| **Studies on peri-implantitis** | |
| Hentenaar et al. 2017  Karring et al. 2004  Rakasevic et al. 2016  Levin et al. 2015  Roos-Jansaker et al. 2015  Büchter et al. 2004  De Waal et al. 2021  Hentenaar et al. 2021 | Follow-up period < 6 months (reporting on peri-implantitis treatment) |
| Arab et al. 2016 | < 5 patients per treatment group |
| Roccuzzo et al. 2011, 2017, 2020 | Compared outher factors than different treatment protocols |
| Alqahtani et al. 2020 | Results presented only graphically |
| Mettraux et al. 2015  Nart et al. 2020  Stein et al. 2017 | Lack of a control/comparative treatment group |
| Schlee et al. 2019 | Did not report on clinical treatment outcomes |

[1-28]

1. Abduljabbar, T., *Effect of mechanical debridement with and without adjunct antimicrobial photodynamic therapy in the treatment of peri-implant diseases in prediabetic patients.* Photodiagnosis Photodyn Ther, 2017. **17**: p. 9-12.

2. Alqahtani, F., et al., *Efficacy of mechanical debridement with and without adjunct antimicrobial photodynamic therapy in the treatment of peri-implantitis among moderate cigarette-smokers and waterpipe-users.* Photodiagnosis Photodyn Ther, 2019. **28**: p. 153-158.

3. Alqahtani, F., et al., *Efficacy of Nonsurgical Mechanical Debridement With and Without Adjunct Low-Level Laser Therapy in the Treatment of Peri-Implantitis: A Randomized Controlled Trial.* J Oral Implantol, 2020. **46**(5): p. 526-531.

4. Alzoman, H., et al., *Comparison of an Herbal- and a 0.12% Chlorhexidine-based Oral Rinse as Adjuncts to Nonsurgical Mechanical Debridement in the Management of Peri-implant Mucositis: A Randomised Controlled Trial.* Oral Health Prev Dent, 2020. **18**(1): p. 645-651.

5. Arab, H., et al., *Comparison of Two Regenerative Surgical Treatments for Peri-Implantitis Defect using Natix Alone or in Combination with Bio-Oss and Collagen Membrane.* J Long Term Eff Med Implants, 2016. **26**(3): p. 199-204.

6. Büchter, A., et al., *Sustained release of doxycycline for the treatment of peri-implantitis: randomised controlled trial.* Br J Oral Maxillofac Surg, 2004. **42**(5): p. 439-44.

7. De Siena, F., et al., *Adjunctive glycine powder air-polishing for the treatment of peri-implant mucositis: an observational clinical trial.* Int J Dent Hyg, 2015. **13**(3): p. 170-6.

8. de Tapia, B., et al., *Adjunctive effect of modifying the implant-supported prosthesis in the treatment of peri-implant mucositis.* J Clin Periodontol, 2019. **46**(10): p. 1050-1060.

9. Flichy-Fernández, A.J., et al., *The effect of orally administered probiotic Lactobacillus reuteri-containing tablets in peri-implant mucositis: a double-blind randomized controlled trial.* J Periodontal Res, 2015. **50**(6): p. 775-85.

10. Hallström, H., S. Lindgren, and S. Twetman, *Effect of a chlorhexidine-containing brush-on gel on peri-implant mucositis.* Int J Dent Hyg, 2017. **15**(2): p. 149-153.

11. Hentenaar, D.F.M., et al., *Erythritol air-polishing in the non-surgical treatment of peri-implantitis; a randomized controlled trial.* Clin Oral Implants Res, 2021.

12. Hentenaar, D.F.M., et al., *Implant decontamination with phosphoric acid during surgical peri-implantitis treatment: a RCT.* Int J Implant Dent, 2017. **3**(1): p. 33.

13. Karring, E.S., et al., *Treatment of peri-implantitis by the Vector system.* Clin Oral Implants Res, 2005. **16**(3): p. 288-93.

14. Kashefimehr, A., et al., *Effects of enamel matrix derivative on non-surgical management of peri-implant mucositis: a double-blind randomized clinical trial.* Clin Oral Investig, 2017. **21**(7): p. 2379-2388.

15. Levin, L., et al., *Water jet with adjunct chlorhexidine gel for nonsurgical treatment of peri-implantitis.* Quintessence Int, 2015. **46**(2): p. 133-7.

16. Mettraux, G.R., et al., *Two-year clinical outcomes following non-surgical mechanical therapy of peri-implantitis with adjunctive diode laser application.* Clin Oral Implants Res, 2016. **27**(7): p. 845-9.

17. Nart, J., et al., *Non-surgical therapeutic outcomes of peri-implantitis: 12-month results.* Clin Oral Investig, 2020. **24**(2): p. 675-682.

18. Rakašević, D., et al., *Efficiency of photodynamic therapy in the treatment of peri-implantitis – A three-month randomized controlled clinical trial.* Srp Arh Celok Lek, 2016. **144**(9-10): p. 478-84.

19. Roccuzzo, M., et al., *Surgical therapy of peri-implantitis lesions by means of a bovine-derived xenograft: comparative results of a prospective study on two different implant surfaces.* J Clin Periodontol, 2011. **38**(8): p. 738-45.

20. Roccuzzo, M., et al., *Implant survival after surgical treatment of peri-implantitis lesions by means of deproteinized bovine bone mineral with 10% collagen: 10-year results from a prospective study.* Clin Oral Implants Res, 2020. **31**(8): p. 768-776.

21. Roccuzzo, M., et al., *Surgical treatment of peri-implantitis intrabony lesions by means of deproteinized bovine bone mineral with 10% collagen: 7-year-results.* Clin Oral Implants Res, 2017. **28**(12): p. 1577-1583.

22. Roos-Jansåker, A.M., U.S. Almhöjd, and H. Jansson, *Treatment of peri-implantitis: clinical outcome of chloramine as an adjunctive to non-surgical therapy, a randomized clinical trial.* Clin Oral Implants Res, 2017. **28**(1): p. 43-48.

23. Stein, J.M., C. Hammächer, and S.S. Michael, *Combination of ultrasonic decontamination, soft tissue curettage, and submucosal air polishing with povidone-iodine application for non-surgical therapy of peri-implantitis: 12 Month clinical outcomes.* J Periodontol, 2017.

24. Strooker, H., S. Rohn, and A.J. Van Winkelhoff, *Clinical and microbiologic effects of chemical versus mechanical cleansing in professional supportive implant therapy.* Int J Oral Maxillofac Implants, 1998. **13**(6): p. 845-50.

25. Tenore, G., et al., *Evaluation of adjunctive efficacy of diode laser in the treatment of peri-implant mucositis: a randomized clinical trial.* Lasers Med Sci, 2020. **35**(6): p. 1411-1417.

26. Schenk, G., et al., *Controlled local delivery of tetracycline HCl in the treatment of periimplant mucosal hyperplasia and mucositis. A controlled case series.* Clin Oral Implants Res, 1997. **8**(5): p. 427-33.

27. González-Serrano, J., et al., *Short-term efficacy of a gel containing propolis extract, nanovitamin C and nanovitamin E on peri-implant mucositis: A double-blind, randomized, clinical trial.* J Periodontal Res, 2021.

28. Schlee, M., et al., *Treatment of Peri-implantitis-Electrolytic Cleaning Versus Mechanical and Electrolytic Cleaning-A Randomized Controlled Clinical Trial-Six-Month Results.* J Clin Med, 2019. **8**(11).
