# Supplementary material for: Efficacy of alternative or adjunctive measures to conventional non-surgical and surgical treatment of peri-implant mucositis and peri-implantitis: a systematic review and meta-analysis
Source: Int J Implant Dent. 2021 Nov 15;7:112. doi: 10.1186/s40729-021-00388-x (PMC8593130; doi:10.1186/s40729-021-00388-x)
Supplement: Supplementary file 2 — Additional file 2: a. Risk‐of‐bias summary of included randomized trial according to the RoB2 risk of bias tool for randomized studies. b. Risk‐of‐bias summary according to the ROBINS-I risk of bias tool for non-randomized studies. [file 40729_2021_388_MOESM2_ESM.docx]

**Supplement 2**

**Supplement 2a.** Risk‐of‐bias summary of included randomized trial according to the RoB2 risk of bias tool for randomized studies

| **Publication** | **Random sequence generation** | **Allocation concealment** | **Blinding of participants and personnel** | **Blinding of outcome assessment** | **Incomplete outcome data** | **Selective reproting** | **Other bias** | **Overall** |
| --- | --- | --- | --- | --- | --- | --- | --- | --- |
| **Studies reporting on peri-implant mucositis treatment** | | | | | | | | |
| Alternative measures for biofilm removal | | | | | | | | |
| Ji et al. 2014 | + | ? | - | + | + | + | + | ? |
| Riben-Grundstrim et al. 2015 | + | + | - | + | + | + | + | - |
| Wohlfahrt et al. 2018 | - | - | + | + | + | + | + | - |
| Adjunctive diode laser/antimicrobial photodynamic therapy | | | | | | | | |
| Javed et al. 2017 | ? | + | ? | ? | - | - | - | ? |
| Al Rifaiy et al. 2018 | ? | + | ? | + | - | - | - | ? |
| Aimetti et al 2019/ Mariani et al. 2020 | + | + | + | + | + | + | + | + |
| Deeb et al. 2020 | ? | + | - | - | + | + | + | ? |
| Adjunctive local antiseptics/systemic antibiotics | | | | | | | | |
| Porras et al. 2002 | ? | - | - | + | - | + | + | ? |
| Thöne-Mühling et al. 2010 | - | ? | - | - | + | + | + | ? |
| Hallström et al. 2012 | + | ? | - | - | + | + | + | ? |
| Menez et al. 2016 | + | + | ? | ? | + | + | + | ? |
| Iorio-Siciliano et al. 2020 | + | + | + | + | + | + | + | + |
| Adjunctive probiotics | | | | | | | | |
| Pena et al. 2019 | + | + | + | + | + | + | + | + |
| Galofre et al. 2018 | + | + | + | + | + | + | + | + |
| Adjunctive antiseptic home care mouthrinse | | | | | | | | |
| Pulcini et al. 2019 | + | + | + | + | + | + | + | + |
| Bunk et al. 2020 | + | + | + | + | + | + | + | + |
| Philip et al. 2020 | - | + | + | + | + | + | + | - |
| **Studies reporting on non-surgical treatment of peri-implantitis** | | | | | | | | |
| Alternative measures for biofilm removal | | | | | | | | |
| Schwarz et al. 2005 | + | - | - | + | + | + | + | - |
| Schwarz et al. 2006 | + | - | - | + | + | + | + | - |
| Renvert et al. 2009 | + | ? | - | + | + | + | + | ? |
| Renvert et al. 2011 | + | - | - | + | + | + | + | - |
| Sahm et al. 2011, John et al. 2011 | + | - | - | + | + | + | + | - |
| Adjunctive diode laser/aPDT | | | | | | | | |
| Wang et al. 2019 | ? | + | + | + | + | + | + | ? |
| Adjunctive local antiseptics/antibiotics | | | | | | | | |
| Renvert et al. 2006 | + | + | - | - | + | + | + | - |
| Renvert et al. 2008 | + | + | - | + | + | + | + | - |
| Schär et al. 2012, Bassetti et al. 2013 | + | - | - | + | - | + | + | - |
| Machtei et al. 2012 | + | + | ? | + | + | + | + | ? |
| Machtei et al. 2020 | + | + | + | + | + | + | + | + |
| Merli et al. 2020 | + | + | + | + | + | + | + | + |
| Adjunctive systemic antibiotics | | | | | | | | |
| Gomi et al. 2015 | + | ? | ? | ? | + | + | + | ? |
| Adjunctive probiotics | | | | | | | | |
| Tada et al. 2017 | + | + | - | + | + | + | + | + |
| Laleman et al. 2019 | + | + | + | + | + | + | + | + |
| **Studies reporting on surgical treatment of peri-implantitis** | | | | | | | | |
| Adjunctive and alternative measures for implant surface decontamination following non-reconstructive therapy | | | | | | | | |
| Papadopoulos et al. 2015 | + | - | - | + | + | + | + | - |
| Hallström et al. 2017 | + | ? | ? | - | + | + | + | ? |
| Albaker et al. 2018 | + | ? | + | + | + |  |  | ? |
| Toma et al. 2019 | - | + | + | + | + | + | + | - |
| Cha et al. 2019 | + | + | + | + | + | + | + | + |
| De Waal et al. 2013 | + | + | ? | + | + | + | + | ? |
| De Waal et al. 2015 | + | + | ? | + | + | + | + | ? |
| Carcuac et al. 2016, 2017 | - | + | + | + | + | + | + | - |
| Adjunctive implantoplasty following non-reconstructive therapy | | | | | | | | |
| Romeo et al. 2005, 2007 | ? | - | - | - | + | + | + | ? |
| Lasserre et al. 2020 | ? | + | + | + | + |  |  | ? |
| Studies comparing non-reconstructive and resonstructive surgical peri-implantitis treatment | | | | | | | | |
| Wohlfahrt et al. 2012/Andersen et al. 2017 | + | + | - | + | + | + | + | - |
| Hamzacebi et al. 2015 | ? | ? | - | ? | + | ? | - | ? |
| Jepsen et al 2016 | + | + | + | - | + | + | + | - |
| Renvert et al. 2018 | + | + | - | + | + | ? | + | ? |
| Isehed et al. 2016, 2018 | - | + | + | + | + | + | + | - |
| Renvert et al. 2021 | ? | ? | ? | ? | + | + | + | ? |
| Adjunctive and alternative measures for implant surface decontamination following reconstructive therapy | | | | | | | | |
| Isler et al. 2018a | + | + | - | + | + | + | + | + |
| Reconstruction of the defect with different bone fillers, with and without a membrane | | | | | | | | |
| Schwarz et al. 2006, 2008, 2009 | + | - | - | + | + | + | + | - |
| Aghazadeh et al. 2012 | ? | ? | - | + | + | + | + | ? |
| Isler et al. 2018b | + | + | + | + | + | + | + | + |
| Polymeri et al. 2020 | + | + | + | ? | + | + | + | ? |
| Studies reporting combined surgical peri-implantitis treatment | | | | | | | | |
| Schwarz et al. 2011, 2012, 2013, 2017 | + | - | - | + | + | + | + | - |
| De Tapia et al. 2019 | + | + | + | + | + | + | + | + |

Low +; high -; unclear ?

**Supplement 2b.** Risk‐of‐bias summary according to the ROBINS-I risk of bias tool for non-randomized studies.

| **Publication** | **Bias due to confounding** | **Bias due to selection of participants** | **Bias in classification of interventions** | **Bias due to deviations from intended interventions** | **Bias due to missing data** | **Bias in measurement of outcomes** | **Bias in selection of the reported results** | **Overall** |
| --- | --- | --- | --- | --- | --- | --- | --- | --- |
| **Studies reporting on peri-implant mucositis treatment**  Alternative measures for biofilm removal | | | | | | | | |
| De Siena et al. 2014 | * | * | * | + | + | + | + | * |
| **Studies reporting on non-surgical treatment of peri-implantitis**  Adjunctive diode laser/aPDT | | | | | | | | |
| Arisan et al 2015 | * | * | * | + | + | * | + | * |
| Adjunctive systemic antibiotics | | | | | | | | |
| Shibli et al. 2019 | * | * | * | + | + | * | + | * |
| **Studies reporting on surgical peri-implantitis treatment**  Adjunctive and alternative measures for implant surface decontamination following reconstructive therapy | | | | | | | | |
| Deppe et al. 2007 | * | * | * | + | * | * | + | * |
| Reconstruction of the defect with different bone fillers, with and without a membrane | | | | | | | | |
| Khoury et al. 2001 | * | * | * | + | ! | ! | + | ! |
| Roos-Jansaker et al. 2007, 2011, 2014 | * | * | * | + | * | * | + | ! |
| Güler et al. 2016 | * | * | ! | + | + | + | + | ! |

Critical !; serious *; moderate -; low+
